# MassSpecBlocks: a web-based tool to create building blocks and sequences of nonribosomal peptides and polyketides for tandem mass spectra analysis

**DOI:** 10.1186/s13321-021-00530-2

**Published:** 2021-07-07

**Authors:** Jan Přívratský, Jiří Novák

**Affiliations:** 1 Faculty of Information Technology, Czech Technical University in Prague, Thákurova 9, 160 00 Prague, Czech Republic; 2 Institute of Microbiology, Czech Academy of Sciences, Vídeňská 1083, 142 20 Prague, Czech Republic

**Keywords:** Mass spectrometry, Building blocks, Nonribosomal petides, Polyketides, Siderophores, MassSpecBlocks, CycloBranch, Tanimoto similarity, SmilesDrawer

## Abstract

Nonribosomal peptides and polyketides are natural products commonly synthesized by microorganisms. They are widely used in medicine, agriculture, environmental protection, and other fields. The structures of natural products are often analyzed by high-resolution tandem mass spectrometry, which becomes more popular with its increasing availability. However, the characterization of nonribosomal peptides and polyketides from tandem mass spectra is a nontrivial task because they are composed of many uncommon building blocks in addition to proteinogenic amino acids. Moreover, many of them have cyclic and branch-cyclic structures. Here, we introduce MassSpecBlocks – an open-source and web-based tool that converts the input chemical structures in SMILES format into sequences of building blocks. The structures can be searched in public databases PubChem, ChemSpider, ChEBI, NP Atlas, COCONUT, and Norine and edited in a user-friendly graphical interface. Although MassSpecBlocks can serve as a stand-alone database, our primary goal was to enable easy construction of custom sequence and building block databases, which can be used to annotate mass spectra in CycloBranch software. CycloBranch is an open-source, cross-platform, and stand-alone tool that we recently released for annotating spectra of linear, cyclic, branched, and branch-cyclic nonribosomal peptides and polyketide siderophores. The sequences and building blocks created in MassSpecBlocks can be easily exported into a plain text format used by CycloBranch. MassSpecBlocks is available online or can be installed entirely offline. It offers a REST API to cooperate with other tools.

**Supplementary Information:**

The online version contains supplementary material available at 10.1186/s13321-021-00530-2.

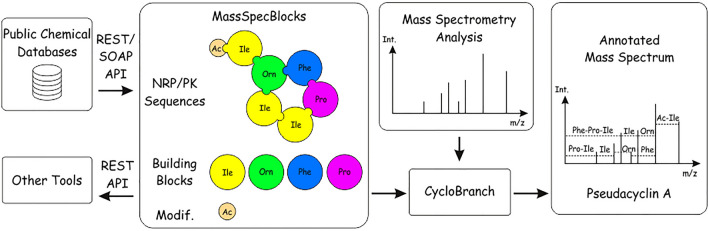

## Introduction

Nonribosomal peptides (NRPs) are natural products commonly produced by bacteria and fungi [1]. NRPs have a wide range of use in human and veterinary medicine, agriculture, the food industry, environmental protection, and other fields. They serve as antibiotics, immunosuppressants, cytostatics, toxins, surfactants, siderophores, etc. The siderophores [2, 3] are iron carriers and promising markers of infectious diseases caused, e.g., by *Aspergillus fumigatus* or *Pseudomonas aeruginosa* [4, 5]. While ribosomal peptides are described by linear sequences of amino acid residues (i.e., monomers or building blocks), NRPs frequently form cyclic and branch-cyclic structures. In contrast to ribosomal peptides, the sequences of NRPs consist of various building blocks, including proteinogenic and non-proteinogenic amino acids, N- and C-methylated residues, N-formylated residues, hydroxy acids, residues with N-terminally attached fatty acid chains, chromophores, and many others [6]. Many siderophores are not peptides but polyketides (PKs) [3, 7].

Natural products are commonly analyzed using mass spectrometry [8]. Molecules in a sample are ionized and separated by mass-to-charge (*m/z*) ratios. Mass spectra contain *m/z* ratios and intensities of collected ions or their fragments in the case of tandem mass spectrometry. The fragmentation principles of peptides have been described many times in the literature [9]. If collision-induced dissociation is used as a fragmentation technique, the spectra of linear peptides commonly contain series of y, b, and a-ions. Because a ring of a cyclic peptide can theoretically be opened between any two consecutive building blocks prior to fragmentation, we observe multiple overlapping series of b-ions in the spectra of cyclic peptides [6].

**Fig. 1:**
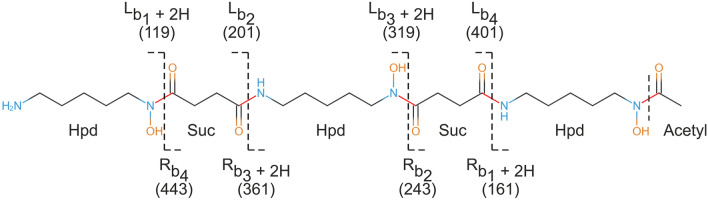
The structure, building blocks, and fragmentation of linear polyketide siderophore desferrioxamine B. The nominal masses of fragment ions are reported. Because the molecule has two N-termini, we use the letters L (left) and R (right) to distinguish between the two series of b-ions

In this work, we focus on high-resolution tandem mass spectra of NRPs and siderophores with NRP/PK structures [6, 7]. An automated interpretation of NRP mass spectra still remains a challenging task due to the complex structures of the molecules. Moreover, PK siderophores contain uncommon building blocks that turn over the orientation of peptide bonds in molecules (e.g., desferrioxamines or ornibactins) and further complicate mass spectra interpretation [7]. For example, desferrioxamine B is composed of two types of building blocks Hpd (N-hydroxy-1,5-pentanediamine) and Suc (succinic semialdehyde) which alternate regularly (Fig. 1). In consequence, the molecule has two N-termini, and uncommon series of ions arise during mass spectra analysis. The observed peaks correspond to b-ions and ions whose masses are increased by the mass of two hydrogens [7].

The spectra of natural products are commonly annotated using database search. Recent advances in microbial chemistry give rise to various databases of natural products. For example, the Natural Products Atlas [10] collects microbial natural products published in peer-reviewed scientific literature. At the time of writing this paper, it contained over 29 thousand compounds. COCONUT [11] is another database collecting data from more than 50 resources and has almost 407 thousand unique natural products. Also, the sizes of other chemical databases grow rapidly. In May 2021, the PubChem database contained about 110 million [12], ChemSpider over 100 million [13], and ChEBI over 59 thousand compounds [14].

The process of determining already known compounds is referred to as dereplication. Several web-based tools have been described for the dereplication of NRPs from tandem mass spectra. An early tool was iSNAP, which included a built-in database containing about 1100 NRP structures compiled from Antibase and the Dictionary of Natural Products [15, 16]. Users could upload a text file containing custom structures in SMILES format [17, 18]. They could define sites where a theoretical structure was fragmented, e.g., peptide and ester bonds. Input experimental spectra were then compared with theoretical spectra. The Dereplicator [19] is a recent tool accessible on GNPS platform [20] that allows the annotation of known peptidic natural products using an in silico fragmentation tree. Two other alternatives of Dereplicator have also been presented. The Dereplicator+ [21] which enables the annotation of non-peptidic natural products, and Dereplicator VarQuest [22], which allows modification-tolerant database search of mass spectra.

NRPro [23] is another recently released tool that compares the input experimental spectra with theoretical spectra generated from the sequences of building blocks, which are available in Norine database [24]. The database of NRPs and their building blocks was released in 2008. Nowadays, it contains 544 blocks and 1740 structures of NRPs composed of these monomers [25]. Users can contribute to the database with missing NRPs and monomers using the interface called MyNorine [26]. The monomeric structures of NRPs can be created using Smiles2Monomers [27] or rBAN [28] tools. However, users cannot create custom databases of building blocks and NRP sequences. Moreover, it’s not always easy to export flawless sequences of building blocks for tandem mass spectra analysis.

An alternative approach for NRP mass spectra interpretation is de novo sequencing [6]. In 2015, we released CycloBranch – an open-source, cross-platform, and stand-alone tool implemented in C++ and dedicated to de novo analysis and dereplication of linear, cyclic, branched, and branch-cyclic NRPs from tandem mass spectra [6, 29, 30]. The de novo sequencing was based on an input database of NRP building blocks. A graph was created from an experimental spectrum where vertices corresponded to *m/z* values. An edge was inserted into the graph if a difference between any two *m/z* values fitted a mass of a block or combination of more blocks (pairs, triplets, etc.). The graph was then browsed starting from an artificial vertex corresponding to the *m/z* value of H+ or H3O+ ion if b-ion or y-ion series were searched, respectively. The final vertex corresponded to the *m/z* value of the precursor ion. NRP sequence candidates were generated from the paths assigned to b-ion or y-ion series. Their theoretical spectra were then compared with the experimental spectrum and sorted using a scoring function. The dereplication was also supported but required an input database of sequences composed of NRP building blocks. In 2017, CycloBranch was extended to support the de novo sequencing and dereplication of NRP/PK siderophores from tandem mass spectra [7]. We also added the ability for dereplication and de novo analysis of natural products in conventional, liquid-chromatography, and imaging mass spectra using fine structures of isotopic peaks [7, 31]. Recently, CycloNovo was also released for de novo sequencing of cyclic peptides [32]. The tool is based on the construction of de Bruijn graphs from experimental spectra and the prediction of *k*-mers (short sequences composed of *k* amino acids). Although the algorithm was designed for cyclic peptides composed of proteinogenic amino acids, it was also successfully applied to NRPs. While CycloBranch is focused on single spectra analysis, CycloNovo was designed for high-throughput analysis of mass spectra and is available on the GNPS platform [20]. A comprehensive overview of other related approaches has been reported by Mohimani and Pevzner [33].

**Fig. 2:**
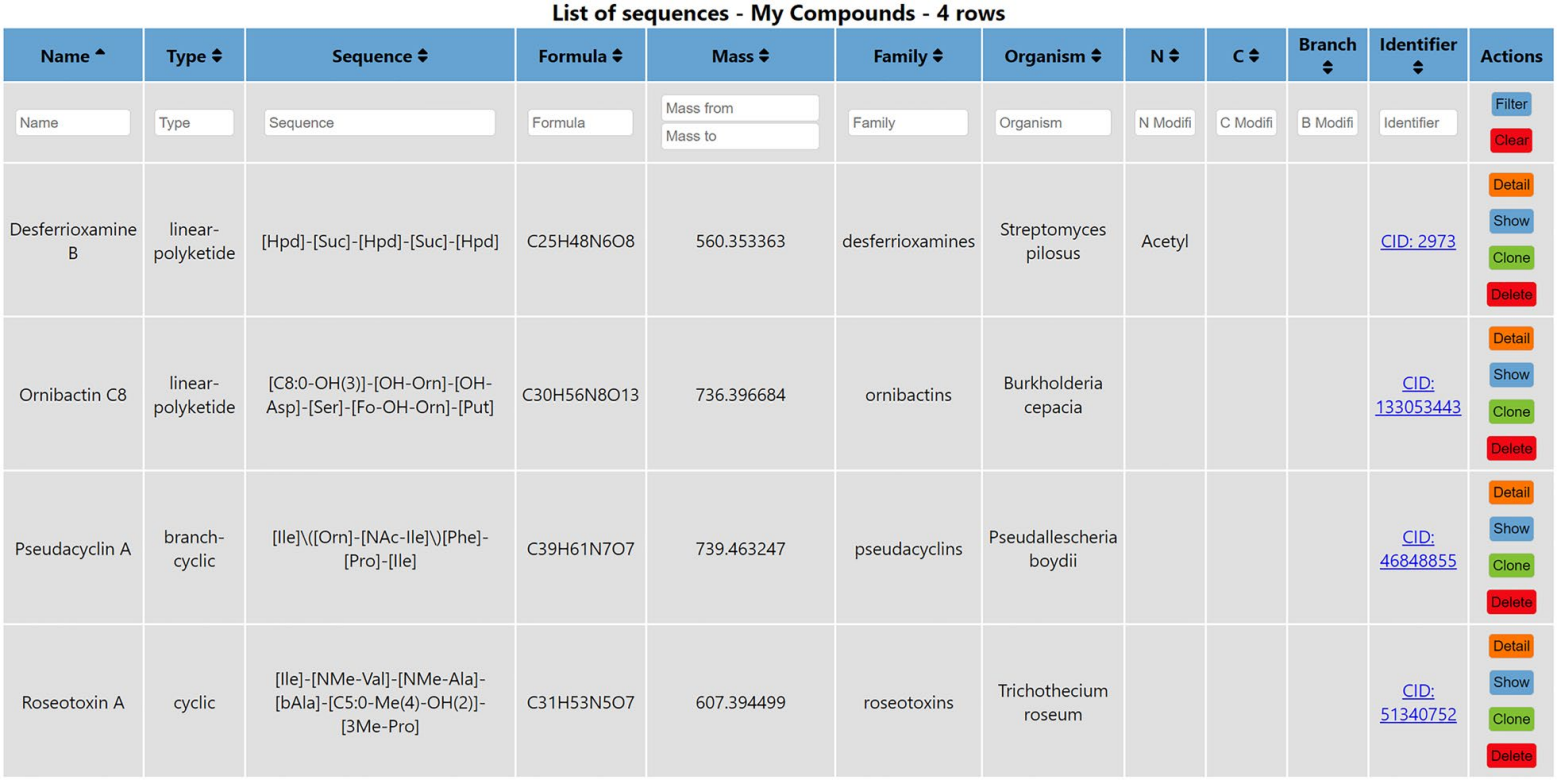
Sample list of NRP and PK sequences

Here, we present MassSpecBlocks [34]—an open-source and web-based tool for easy construction of NRP/PK sequence (Fig. 2) and building block (Additional file 1: Fig. S1) databases, which can be used in CycloBranch software to compare the experimental tandem mass spectra of NRPs/PKs with theoretical spectra [6, 7]. Although the databases of building blocks and sequences can be created directly in CycloBranch, the process requires a lot of manual labor. The structures of compounds must be searched in a database by a web browser. Residue formulas of building blocks must be calculated manually from structural formulas. Acronyms of building blocks, their residue formulas, and sequences of building blocks must be typed manually into CycloBranch. MassSpecBlocks allows automatization of this process. Anyone can create custom databases of NRP/PK sequences and their building blocks in a user-friendly graphical interface. The databases can be shared with collaborators and easily exported into a plain text format to be used for mass spectra analysis in CycloBranch software. The molecular structures in SMILES format can be searched in PubChem, ChemSpider, ChEBI, NP Atlas, COCONUT, and Norine databases covering a wide range of known compounds. MassSpecBlocks is freely available at https://ms.biomed.cas.cz/msb and can be installed entirely offline on a local computer. The tool provides a REST API to access the data. The source codes and installation instructions are available on GitHub [35].

## Implementation

**Fig. 3:**
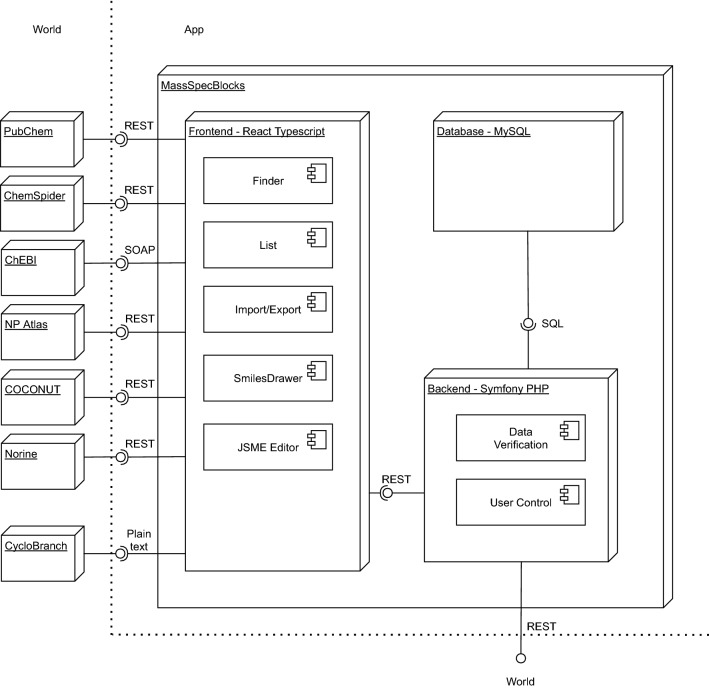
Architecture of MassSpecBlocks

The architecture of MassSpecBlocks is shown in Fig. 3. The front-end has been implemented in TypeScript using the JavaScript library React (v. 16.13.1) [36] and Node.js runtime environment (v. 15.14.0) [37]. It becomes a common standard that chemical databases offer an Application Programming Interface (API) to access their data. PubChem [38], ChemSpider [39], NP Atlas [40], COCONUT [11], and Norine [26] provide REST (Representational State Transfer) APIs, while the ChEBI database has SOAP (Simple Object Access Protocol) API [41]. MassSpecBlocks utilizes these APIs to collect the data about chemical structures using the *Finder* component. The range of supported databases can be extended if a new class implementing the *Finder*’s interface is created. The *List* component is used to obtain the data from a local database and visualize it in interactive tables, which can be edited using a mouse-click on an individual item. The compounds are drawn using SmilesDrawer (a modified version forked from v. 1.2.0) [42]. A disadvantage of SmilesDrawer was that the structures could not be edited in graphics mode. Thus we used a freely available JavaScript Molecule Editor JSME (v. 2017-02-26) to support this feature [43].

The database is stored in MySQL [44], and the backend has been implemented in PHP framework Symfony (v. 5.2) [45]. It provides a REST API to access the data, takes care of the user access rights, data verification and transformation into SQL queries, etc. The REST API can also be used to cooperate with other tools. The documentation is available on our server [46] or at https://localhost:*port*/rest/doc if the backend is installed on a local machine, where the *port* is a number of the port where the server is running. The API currently has 77 endpoints to manipulate the database. The individual endpoints can be tested directly from the documentation page. The results are returned in JSON file format. Alternatively, the data can be exported into plain text files accepted by CycloBranch software.

### Data model

The data model includes entities representing building blocks, sequences, and terminal modifications of sequences (Additional file 1: Fig. S2). Sequences and building blocks with similar structures are grouped into families. A list of organisms can be assigned to a sequence. Every entity has a relationship with a container (Additional file 1: Fig. S3). The container groups data (sequences, blocks, modifications, etc.) owned by a user. A registered user can create/edit multiple private containers, which can be cloned and shared with collaborators (Additional file 1: Fig. S4). An administrator can also create public containers. An unregistered user can access compounds in public containers and can search in PubChem, ChEBI, NP Atlas, COCONUT, and Norine. The registered user can also search in ChemSpider if an API key is provided. The data in containers can be exported into (or imported from) a plain text format accepted by CycloBranch software (i.e., lists of sequences, blocks, and modifications). The default installation includes three public containers: (1) *Nonribosomal Peptides and Siderophores* is a sample container that includes 85 building blocks and 146 sequences of amphibactins, aquachelins, beauverolides, cyclosporins, desferrioxamines, ferrichromes, fusarinines, gramicidins, ornibactins, pseudacyclins, pyoverdines, roseotoxins, etc. (2) The container *Proteinogenic Amino Acids* includes the twenty basic building blocks. It can be cloned and used for user purposes. (3) *Siderophores and Secondary Metabolites (MS)* is a container that includes a list of 709 compounds taken from the reference [2]. The last container does not include the sequences of building blocks and can be used by CycloBranch software to detect whole compounds in conventional, liquid-chromatography, and imaging mass spectrometry data [7, 31].

### Frontend

**Fig. 4:**
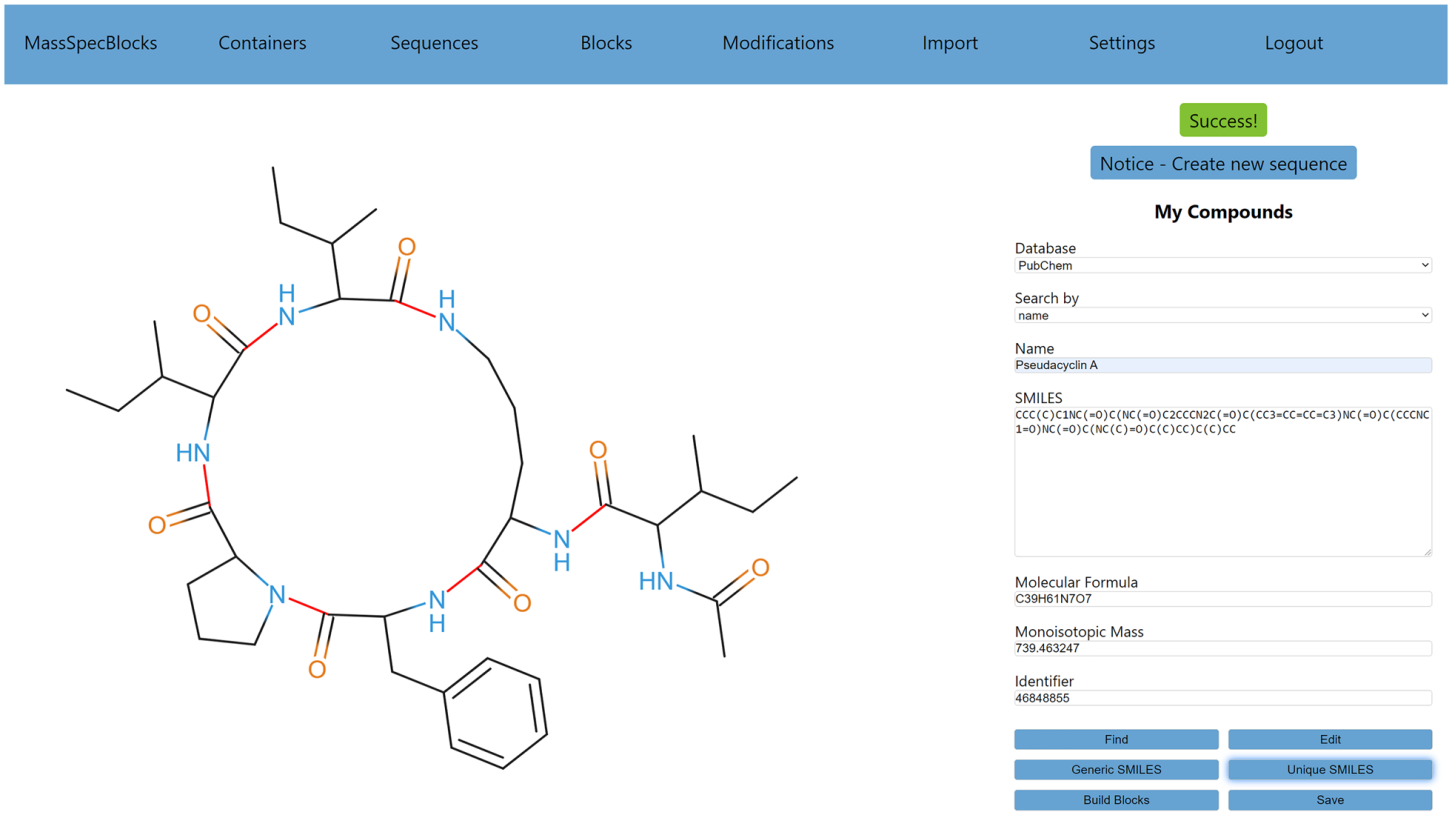
Annotation of peptide bonds in the structure of pseudacyclin A

**Fig. 5:**
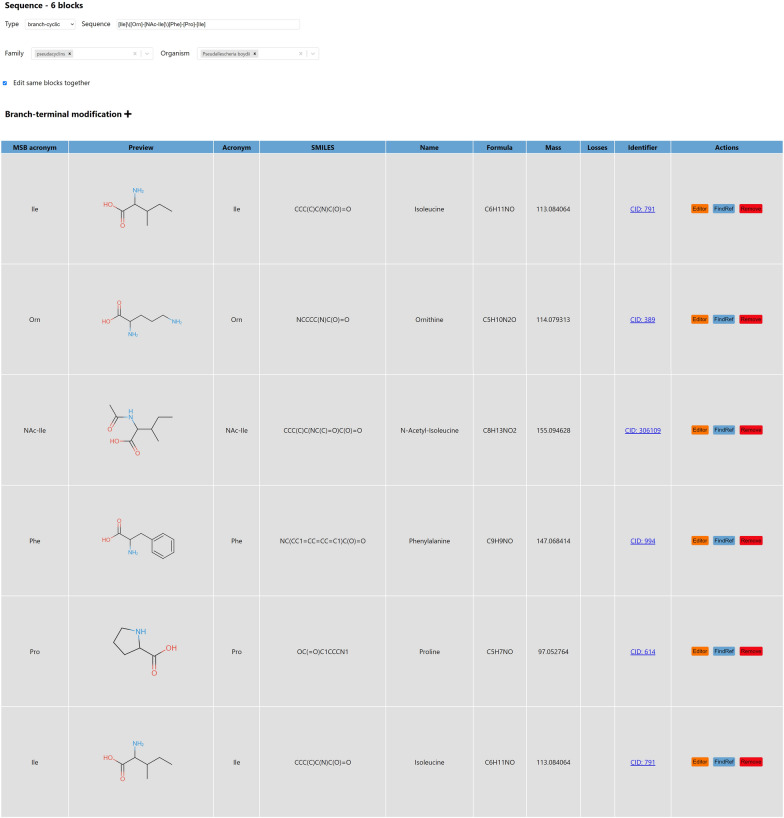
Building blocks of pseudacyclin A

The main screen of MassSpecBlocks is shown in Fig. 4. Users can search compounds in public databases using one of the following properties—a name, a molecular formula, a monoisotopic mass, a database identifier, and a molecular structure in SMILES format (if the corresponding API supports the respective feature). The *Find* button starts a search process. Missing properties in the input form are filled automatically when the process is completed. The molecular structure can be edited by a direct change of the SMILES notation or using the *Edit* button, which opens JSME. The SMILES format can handle markups that are used in stereochemistry, but this information cannot commonly be utilized in mass spectra analysis. Thus the button *Generic SMILES* removes these markups from the notation. Because a structure in SMILES format can be defined in many ways, another button *Unique SMILES* turns the notation into its canonical form using the algorithms CANON and GENES described by Weininger, D. et al. [47]. A list of building blocks is generated using the button *Build Blocks* (Fig. 5). The SMILES strings of building blocks are searched in a local database first (i.e., in a selected container) to obtain the remaining properties of the blocks. If a string is not present in the local database, the metadata is searched in PubChem. When a user checks the results, the sequence, blocks, and remaining metadata can be stored in the current container using the *Save* button.

#### Building blocks and sequences

The other pages in MassSpecBlocks are dedicated to the administration of building blocks (Additional file 1: Fig. S1), sequences (Fig. 2), terminal modifications (Additional file 1: Fig. S5), containers (Additional file 1: Fig. S3) and account settings. A building block has the following properties: a name, a unique acronym, molecular formula and monoisotopic mass of residue, a list of neutral losses, a family, and a reference to a public database. The monoisotopic mass is not mandatory because it is always calculated from the formula. The list of neutral losses can include multiple formulas separated by a semicolon (e.g. ”H2O;NH3”). A reference to an external database is composed of a database identifier and an identifier of a referenced item. For example, alanine can be referenced by ”CID: 602”, ”CSID: 582”, and ”CHEBI: 16449” in PubChem, ChemSpider, and ChEBI databases, respectively.

NRP/PK sequences have the following properties: a compound name, a structure type (*linear*, *cyclic*, *branched*, *branch-cyclic*, *linear-polyketide*, *cyclic-polyketide*, and *other*), a sequence of building blocks, a formula and monoisotopic mass of the neutral molecule, lists of families and organisms, terminal modifications, and a reference into a public database. The format of sequence notation was taken from the CycloBranch software. The sequences of linear and cyclic NRPs/PKs are written in the format [A]-[B]-[C]-[D]-[E], where A to E are acronyms of building blocks. In the case of cyclic NRPs/PKs, we assume that block E is connected to block A. The sequences of single branched and branch-cyclic NRPs have the notation [A]$\backslash$([B]-[C]$\backslash$)[D]-[E], where B is the branching block (e.g., ornithine in pseudacyclin A [6, 48]) and C is the last block of the branch. In the case of branch-cyclic NRPs, E is connected to A. The tool automatically determines the sequence type from the molecular structure. Multiply branched NRPs are automatically annotated as the type *other* due to the data compatibility with CycloBranch software, which currently does not support multiply branched peptides in this notation. Depending on the type of analyzed NRP/PK, we can optionally define the names of up to three terminal modifications. In this case, the modifications must not be included in the respective building blocks and must be defined separately. For example, if a terminal block corresponds to N-acetyl-isoleucine, we can replace the block with isoleucine and define the acetylation as an N-terminal modification (i.e., C2H2O, +42.010565 Da). Every modification is defined using a name (or an acronym), a formula, a monoisotopic mass, and the information whether the modification is N-terminal or C-terminal (Additional file 1: Fig. S5). While linear NRPs may have N-terminal and C-terminal modifications, cyclic NRPs do not have any terminal modifications. A branch modification (possibly N-terminal or C-terminal) may be defined for single branched and branch-cyclic NRPs. A linear PK may have two modifications of the same type (e.g., N-terminal). Note that due to the compatibility with CycloBranch software, the tool does not deal with internal modifications of building blocks. From principle, we define the modified building blocks as new blocks.

#### Families and organisms

MassSpecBlocks automatically recommends families corresponding to the analyzed structures. A premise is that a similar structure is already defined in a container, and a family is assigned to it. The family can be created and assigned to the sequence in the *Family* menu if the new sequence is being created (Fig. 5). Optionally, it can be defined in the list of sequences (Fig. 2) or container configuration (Additional file 1: Fig. S4). The families are recommended using a simple text search or Tanimoto similarity. The administrator can select the model used by the application in the web interface. The text similarity was the first model we implemented, and it was preserved for historical reasons for its simplicity. A substring *S* of a compound name was compared with the names of compounds in the current container. The families corresponding to matched names were recommended. For example, we defined pseudacyclin A and assigned the family name pseudacyclins to it. If the sequence of pseudacyclin B was being created, the substring *S* = “pseudacyclin” was taken from the name pseudacyclin B. Because the substring *S* was found in the sequence name pseudacyclin A, the family pseudacyclins was recommended.

The second approach works the same way with the difference that we do not pair the names of compounds, but we pair their structures using Tanimoto similarity. The Tanimoto similarity [49] is calculated as $T(A,B) = \frac{|A \cap B|}{|A \cup B|} = \frac{|A \cap B|}{|A|+|B|-|A \cap B|}$, where *A* and *B* are the sets of blocks in the respective structures. For example, pseudacyclin A is composed of six building blocks (Figs. 2 and 4). In pseudacyclin B, one isoleucine is substituted by valine [48]. The similarity of these two pseudacyclins is $T(A,B) = \frac{5}{5+6-5} = \frac{5}{6}$. If we have a sequence and search for similar sequences in a container using Tanimoto similarity, we can get multiple results with the same similarity. In this case, a sequence with the smallest difference in the total number of blocks is selected, and the corresponding family is recommended. If more than one family is assigned to the sequence, all the families are recommended. For example, we create the sequence of desferrioxamine B and assign two families, ferrioxamines and siderophores, to it. If we would like to add a new sequence of desferrioxamine E, both families are recommended. Currently, the Tanimoto similarity is used on our web server.

A list of organisms can be assigned to the sequence in the *Organism* menu (Fig. 5). Similar to families, it can also be defined in the list of sequences (Fig. 2) or container configuration (Additional file 1: Fig. S4). However, the organisms are not automatically recommended if the button *Build Blocks* is pressed.

#### SmilesDrawer

SmilesDrawer is a modern and open-source JavaScript library for parsing and drawing SMILES-encoded molecular structures. It does not require any form of client-server communication, and the rendered images are pretty. For our purpose, we had to make the following modifications in the SmilesDrawer’s code: (1) We had to mark peptide (and ester) bonds in a molecular graph of a compound to generate the building blocks. SmilesDrawer represents a molecular graph as an array of vertices (atoms) and an array of edges (bonds). The latter array was traversed, and edges corresponding to peptide bonds were annotated. If a resulting building block would include three or fewer vertices, the corresponding bond was skipped. Thus the terminal modifications like acetylation did not form independent blocks. (2) Because some molecular structures are complex and the automated annotation of peptide bonds may not always produce the desired result, it is advantageous to let a user control this process before a molecular structure is split into building blocks. For this purpose, a mouse interaction feature was added. After a mouse-click event, the edges of the molecular graph were traversed and checked if they overlapped with the current mouse position. If an edge overlapped with the cursor position, the corresponding bond was un/marked as the red break-up point (Fig. 4). (3) The depth-first search (DFS) was implemented to browse the molecular graph and generate the structures of building blocks in SMILES format. The graph was traversed so that the blocks were generated in the correct order from N-terminus to C-terminus. The SMILES strings were fixed so that carboxyl groups were attached at C-termini instead of formyl groups. Finally, the DFS was used to estimate if the resulting structure was linear, cyclic, branched, branch-cyclic and if it corresponded to an NRP or polyketide. In the beginning, the structure was considered to be linear. If a block was found more than once during the DFS, the structure was marked as cyclic. If some block was connected with more than two neighbors, the structure was marked as branched. If both properties were satisfied, the structure was annotated as branch-cyclic. Because CycloBranch currently supports only singly branched and branch-cyclic NRPs, the structure was marked as the type *other* if multiple branches were detected. An NRP building block has N-terminus and C-terminus. If a building block was found which did not have the C-terminus (for example, see the block Hpd in Fig. 1 having nitrogen atoms on both termini), the structure was marked as a polyketide. As in the case of NRPs, we determined if the polyketide was linear or cyclic.

## Results

**Fig. 6:**
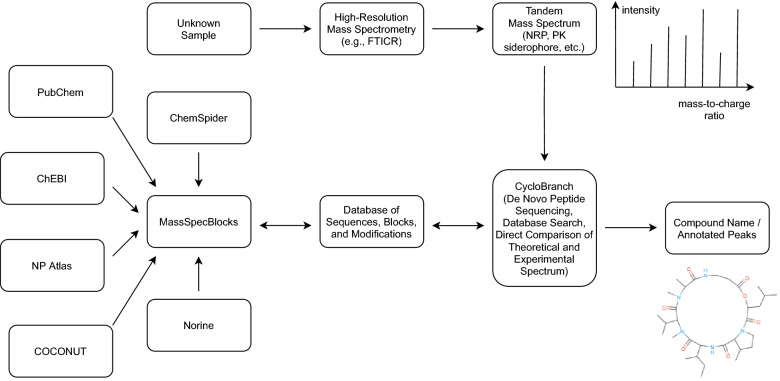
Data processing workflow

In this section, we show a sample decomposition of two NRPs and two linear PK siderophores into building blocks by MassSpecBlocks. We also show how to annotate mass spectra in CycloBranch and comparison of our approach with other methods. The complete data processing workflow is shown in Fig. 6. Further instructions on how to process the data are available in a video tutorial on our website [50]. The databases of sequences, building blocks, and terminal modifications (i.e., the content of a container) were exported from MassSpecBlocks into a simple plain text format (every row in a file corresponds to an item; the values on each row are tab-separated). The high-resolution tandem mass spectra were acquired on a 12T SolariX FTICR mass spectrometer (Bruker Daltonics, Billerica, MA, USA). They are freely available on CycloBranch’s homepage [29] and in a public GNPS library [20]. We used the spectra of pseudacyclin A (CCMSLIB00000531485 in GNPS library), roseotoxin A (CCMSLIB00000531484), ornibactin C8 (CCMSLIB00001059073), desferrioxamine B (CCMSLIB00001059065), and ferrioxamine B (CCMSLIB00001059066). CycloBranch v. 2.0.8 was used to compare the experimental spectra with theoretical spectra generated from the exported databases.

### Nonribosomal peptides

We have chosen NRP pseudacyclin A with the branch-cyclic structure as the first showcase example [6, 48]. The main screen of the MassSpecBlocks application is shown in Fig. 4. The structure of pseudacyclin A in SMILES format and other properties were found in PubChem using the compound name. The peptide is composed of six building blocks. The ring corresponds to the sequence cyclo(Pro-Ile-Ile-Orn-Phe), where Orn stands for ornithine. The last block, Ac-Ile (N-acetyl-isoleucine), is connected to Orn. The tool automatically recognized the peptide bonds and marked them in red. The peptide bond connecting Ac to Ile on the branch was not marked because the resulting block Ac would contain only three nonhydrogen atoms. At this stage, a user could mark or unmark any bond using the computer mouse.

The decomposition of the molecule into building blocks is shown in Fig. 5. MassSpecBlocks correctly determined the branch-cyclic structure of the molecule. The building blocks Ile, Pro, and Phe were found in a local database of building blocks that contained 20 proteinogenic amino acids. Ornithine and N-acetyl-isoleucine were automatically found in PubChem using their structures in SMILES format. Similar to the whole peptide structure, every building block could be modified in JSME using the respective *Edit* button. Finally, the sequence and building blocks were stored in a local database when the *Save* button was pressed. The data was exported from the container into the plain text files using the *Export* button (Additional file 1: Fig. S3). CycloBranch successfully compared the experimental spectrum of pseudacyclin A with the theoretical spectrum generated from the input sequence of building blocks. The sample software configuration is shown in Additional file 1: Fig. S6, and the result of spectra comparison in Additional file 1: Fig. S7.

Another example of annotated peptide bonds and an ester bond in the molecular structure of a depsipeptide roseotoxin A [6, 51] is shown in Additional file 1: Fig. S8. The structure was found in PubChem using the molecular formula C31H53N5O7. MassSpecBlocks listed sixty-six thumbnails of candidate structures. The correct structure of the cyclic peptide was selected manually. The corresponding building blocks are reported in Additional file 1: Fig. S9.

### Polyketide siderophores

In the following example, we have chosen two linear PK siderophores ornibactin C8 and desferrioxamine B. Ornibactins contain C-terminally attached putrescine (Put)—a PK building block that is terminated by amine groups on both sides (Additional file 1: Fig. S10). It’s a common practice that residue formulas of building blocks are calculated using water elimination (e.g., the molecular formula of Ile is C6H13NO2, but the residue formula is C6H11NO). However, because Put does not include any oxygen, the water molecule cannot be eliminated. For this reason, we calculated the residue formula as the elimination of two hydrogens. So, the molecular formula of Put is C4H12N2, but the residue formula is C4H10N2. We can see that MassSpecBlocks correctly determined this building block during the decomposition and annotated ornibactin as the linear polyketide.

The structure of desferrioxamine B and building blocks generated by MassSpecBlocks are shown in Additional file 1: Fig. S11. Analogously to Put, the block Hpd is terminated with amine groups on both sides. Assuming no hydroxyl group is attached to amine in Hpd, the water molecule cannot be eliminated. Thus we define the residue formula of Hpd as C5H12N2O, although the molecular formula is C5H14N2O. The second block Suc has the residue formula C4H4O2 and is terminated by formyl groups on both sides. This leads to the idea that the molecular structure of the block should include carboxyl groups on both sides. Because the exact positions of chemical elements inside a building block cannot be determined by mass spectrometry, we added only one hydroxyl group. So, following the standard definition, we added a water molecule to the residue formula to get the molecular formula of Suc (C4H6O3). We can see that MassSpecBlocks correctly determined the structures of Hpd and Suc. Similar to the previous example, it correctly annotated the structure of desferrioxamine B as the linear polyketide.

The presence of PK building blocks in the molecule impacts its fragmentation, and thus uncommon series of ions arise in mass spectra. The annotated spectra and characteristic fragmentation patterns of ornibactins and desferrioxamines have been discussed in our previous work [7]. Sample configuration of CycloBranch for the spectrum of desferrioxamine B is shown in Additional file 1: Fig. S12. The spectrum was compared with the list of 146 NRP and siderophore sequences in less than a second. In Additional file 1: Fig. S13, we show an output report if a precursor mass filter was enabled. The sequence of desferrioxamine B was reported as a single hit. To show the strength of our approach, we also show a report if the precursor filter was disabled. We can see that the correct sequence was still reported as the top hit. CycloBranch implements several scoring functions, including the number of matched peaks, the sum of relative intensities of matched peaks, the weighted ratio of matched peaks to all peaks (i.e., the sum of relative intensities of matched peaks divided by the sum of intensities of all peaks), etc. In this case, we used the weighted ratio, which was 96.4% for the top hit. The annotated spectrum of desferrioxamine B is shown in Additional file 1: Fig. S14 and was opened by a double-click on the respective row in the output report. We can see that the fragmentation corresponds to those shown in Fig. 1. CycloBranch correctly annotated isotopes of fragment ions because the isolation window used during the mass spectrometry analysis was set up to *m/z* 561.36 ± 2 Da.

### Comparison with other approaches

We used the spectra mentioned above to compare our approach with NRPro (1.0), Dereplicator (1.2.8), and Dereplicator+ (1.0.0). Dereplicator and NRPro were designed mainly for mass spectra annotation of peptidic natural products. These tools visualized the annotated peaks in mass spectra and matching fragments in molecular structures of respective compounds. Dereplicator+ also supported polyketides, but matched fragments were not visualized in molecular structures. CycloBranch used a simplified visualization of building blocks (Additional file 1: Fig. S14). An advantage of NRPro was the ability to retrieve structures missing in Norine from ChEBI, NP Atlas, and PubChem. While Dereplicator and NRPro could analyze spectra only with predefined sodium and potassium adducts (i.e., [M+Na]+ or [M+K]+ ions), CycloBranch offered an ability to annotate spectra with custom adducts, e.g., [M+Fe-2H]+, [M+Fe-3H+Na]+, [M+Fe-3H+K]+, [M+Al-2H]+, etc. Dereplicator+ did not offer us any possibility to define the adducts.

In comparison to other tools, CycloBranch supports annotation of fine structures of isotopic peaks and visualization of profile mass spectra in addition to line spectra. A custom list of neutral losses can be defined in addition to common losses like H2O and NH3. If necessary, additional lists of neutral losses can be defined for individual building blocks. Dereplicator and Dereplicator+ can process the input experimental spectra in mgf, mzXML, mzML file formats. NRPro currently supports mgf and mzXML. CycloBranch support spectra in a plain text file format (txt), in the open formats mgf, mzXML, mzML, and native file formats of Bruker (baf), Waters (raw), and Thermo (raw).

**Table 1:** Comparison with other approaches—the total numbers of peaks in the experimental spectra of NRPs/PKs and the numbers of peaks annotated by the tools. The weighted ratio of matched peaks is also reported for CycloBranch in percent (i.e., the sum of intensities of matched peaks divided by the sum of intensities of all experimental peaks)

NRP/PK	Exp. peaks	CycloBranch	NRPro	Dereplicator	Dereplicator+
Pseudacyclin A	29	26 (99%)	23	6	14
Roseotoxin A	25	21 (97%)	22	8	19
Ornibactin C8	127	31 (47%)	46	7	23
Desferrioxamine B	11	10 (96%)	11	–	–
Ferrioxamine B	42	18 (80%)	–	–	–

The numbers of peaks annotated by the tools are reported in Table 1. The precursor and fragment ion *m/z* error tolerances were set up to 5ppm for CycloBranch and NRPro. The values used in Dereplicator and Dereplicator+ were 0.005 Da. Because the input mgf/mzXML files included line spectra, and the other tools did not offer an option to crop the low-intensity peaks, we set up the minimum threshold of relative peak intensity to zero. To unify the configuration of CycloBranch with NRPro, we were looking only for b-ions, a-ions and allowed only one neutral loss of H2O or NH3. See the sample configuration for pseudacyclin A in Additional file 1: Fig. S6 and desferrioxamine B in Additional file 1: Fig. S12. Because the input databases were different and CycloBranch was a stand-alone tool while the other web-based tools, we could not compare their performance exactly. However, while CycloBranch and NRPro commonly returned results in a second, the average data-processing time was 2 minutes and 4 minutes for Dereplicator and Dereplicator+, respectively.

Pseudacyclin A was reported by all the tools as the single hit. Roseotoxin A was found by CycloBranch and NRPro. NRPro also reported destruxin B1 as an isomer of roseotoxin A whose theoretical peaks fit the same experimental peaks. Dereplicator reported bursaphelocide B (another isomer of roseotoxin A) as the only hit. Dereplicator+ assigned the spectrum to roseocardin whose structure corresponds to roseotoxin A. Ornibactin C8 was reported by all the tools as the most significant hit. The advantage of NRPro was that it also annotated peaks of internal fragment ions, which CycloBranch does not currently support in the case of linear polyketides. On the other hand, CycloBranch marked 55 peaks if we enabled the annotation of peaks with up to three neutral losses. The weighted ratio of matched peaks was 58% in this case. Desferrioxamine B was found by CycloBranch and NRPro. Both tools annotated all the monoisotopic peaks shown in Additional file 1: Fig. S14﻿. CycloBranch was the only tool that annotated the ferri-form of this compound (i.e., the spectrum of [M+Fe-2H]+ ion).

Note that the analysis of desferrioxamines and other PK siderophores requires knowledge of characteristic fragmentation patterns (Fig. 1). Some pre-existing knowledge is also required if the de novo sequencing of NRPs is performed because the combinations of building blocks frequently form isomers. We commonly get many NRP sequence candidates which fit the experimental spectrum well. If we perform the database search, the situation is much easier. Because CycloBranch is focused on the annotation of high-resolution mass spectra, the most important parameters to be tuned are *m/z* error tolerance and the minimum threshold of intensity. The users should also check in the literature which compounds can be produced by organisms they study. Further instructions to fine-tune the tool’s configuration for various use-cases can be found in the Tutorial’s section on the CycloBranch’s website [29] and in our previous work [6, 7, 52].

## Conclusion

MassSpecBlocks was presented as an open platform for creating custom databases of NRPs/PKs where the structures were stored as sequences of building blocks. The tool can be accessed online or installed offline on a local computer; it provides a REST API and can be extended to support the compound search in various public repositories. In combination with the CycloBranch software, we provided a robust workflow for annotating tandem mass spectra of NRPs, PKs, siderophores, and other natural products, which can be represented as sequences of building blocks.

## Supplementary Information

**Additional file 1.** Additional figures.

## Data Availability

The MassSpecBlocks web application can be accessed at https://ms.biomed.cas.cz/msb. The code for MassSpecBlocks is available at https://github.com/privrja/MassSpecBlocks, REST API documentation at https://ms.biomed.cas.cz/msb-backend/public/index.php/rest/doc, and the video tutorial at https://ms.biomed.cas.cz/msb/msb-tutorial.mp4. The modified version of SmilesDrawer is available at https://github.com/privrja/smilesDrawer. Project name: MassSpecBlocks. Project home page: https://github.com/privrja/MassSpecBlocks. Operating system(s): Platform independent. Programming language: TypeScript, JavaScript, PHP, SQL. Other requirements: PHP 7.2.5 or higher, MySQL 8 (MariaDB 10), Node.js 15.14.0. License: MIT. Any restrictions to use by non-academics: Not applicable .

## Abbreviations

API: Application Programming Interface
DFS: Depth First Search
FTICR: Fourier-Transform Ion Cyclotron Resonance
GNPS: Global Natural Products Social Molecular Networking
JSME: JavaScript Molecule Editor
JSON: JavaScript Object Notation
NRP: Nonribosomal Peptide
PHP: Hypertext Preprocessor
PK: Polyketide
REST: Representational State Transfer
SMILES: Simplified Molecular Input Line Entry Specification
SOAP: Simple Object Access Protocol
SQL: Structured Query Language

## References

[1] Strieker M, Tanovic A, Marahiel MA (2010). Nonribosomal peptide synthetases: structures and dynamics. Curr Opin Struct Biol.

[2] Pluhacek T, Lemr K, Ghosh D, Milde D, Novak J, Havlicek V (2016). Characterization of microbial siderophores by mass spectrometry. Mass Spectrom Rev.

[3] Hider RC, Kong X (2010). Chemistry and biology of siderophores. Nat Prod Rep.

[4] Luptakova D, Pluhacek T, Petrik M, Novak J, Palyzova A, Sokolova L, Skriba A, Sediva B, Lemr K, Havlicek V (2017). Non-invasive and invasive diagnoses of aspergillosis in a rat model by mass spectrometry. Sci Rep.

[5] Dobias R, Havlicek V, Das S, Dash HR (2021). Microbial siderophores: markers of infectious diseases. Microbial and natural macromolecules.

[6] Novak J, Lemr K, Schug KA, Havlicek V (2015). CycloBranch: De Novo sequencing of nonribosomal peptides from accurate product ion mass spectra. J Am Soc Mass Spectrom.

[7] Novak J, Sokolova L, Lemr K, Pluhacek T, Palyzova A, Havlicek V (2017). Batch-processing of imaging or liquid-chromatography mass spectrometry datasets and De Novo sequencing of polyketide siderophores. BBA Proteins Proteom.

[8] Prichystal J, Schug KA, Lemr K, Novak J, Havlicek V (2016). Structural analysis of natural products. Anal Chem.

[9] Paizs B, Suhai S (2005). Fragmentation pathways of protonated peptides. Mass Spectrom Rev.

[10] Van Santen, J.A., Jacob, G., Singh, A.L., Aniebok, V., Balunas, M.J., Bunsko, D., Neto, F.C., Castaño-Espriu, L., Chang, C., Clark, T.N., Cleary Little, J.L., Delgadillo, D.A., Dorrestein, P.C., Duncan, K.R., Egan, J.M., Galey, M.M., Haeckl, F.P.J., Hua, A., Hughes, A.H., Iskakova, D., Khadilkar, A., Lee, J.-H., Lee, S., Legrow, N., Liu, D.Y., Macho, J.M., McCaughey, C.S., Medema, M.H., Neupane, R.P., O’Donnell, T.J., Paula, J.S., Sanchez, L.M., Shaikh, A.F., Soldatou, S., Terlouw, B.R., Tran, T.A., Valentine, M., Van Der Hooft, J.J.J., Vo, D.A., Wang, M., Wilson, D., Zink, K.E., Linington, R.G.: The natural products atlas: an open access knowledge base for microbial natural products discovery. ACS Central Sci. **5**(11), 1824–1833 (2019). 10.1021/acscentsci.9b00806 10.1021/acscentsci.9b00806 PMC6891855 31807684

[11] Sorokina M, Merseburger P, Rajan K, Yirik MA, Steinbeck C (2021). COCONUT online: collection of open natural products database. J Cheminformatics.

[12] Kim S, Chen J, Cheng T, Gindulyte A, He J, He S, Li Q, Shoemaker BA, Thiessen PA, Yu B, Zaslavsky L, Zhang J, Bolton EE (2018). PubChem 2019 update: improved access to chemical data. Nucleic Acids Res.

[13] Royal Society of Chemistry: ChemSpider (2021). https://www.chemspider.com/. Accessed 12 Apr 2021

[14] Hastings J, Owen G, Dekker A, Ennis M, Kale N, Muthukrishnan V, Turner S, Swainston N, Mendes P, Steinbeck C (2016). ChEBI in 2016: improved services and an expanding collection of metabolites. Nucleic Acids Res.

[15] Ibrahim A, Yang L, Johnston C, Liu X, Ma B, Magarvey NA (2012). Dereplicating nonribosomal peptides using an informatic search algorithm for natural products (iSNAP) discovery. Proc Natl Acad Sci U S A.

[16] Ibrahim, A., et al.: iSNAP Analogue Search (2021). https://magarveylab.ca/analogue/. Accessed 12 Apr 2021

[17] Weininger D (1988). SMILES, a chemical language and information system. 1. Introduction to methodology and encoding rules. J Chem Inf Comput Sci.

[18] James, C.A.: OpenSMILES specification (2021). http://opensmiles.org/opensmiles.html. Accessed 12 Apr 2021

[19] Mohimani H, Gurevich A, Mikheenko A, Garg N, Nothias L-F, Ninomiya A, Takada K, Dorrestein PC, Pevzner PA (2017). Dereplication of peptidic natural products through database search of mass spectra. Nat Chem Biol.

[20] Wang, M., et al.: GNPS (2021). https://gnps.ucsd.edu/. Accessed 12 Apr 2021

[21] Mohimani H, Gurevich A, Shlemov A, Mikheenko A, Korobeynikov A, Cao L, Shcherbin E, Nothias L-F, Dorrestein PC, Pevzner PA (2018). Dereplication of microbial metabolites through database search of mass spectra. Nat Commun.

[22] Gurevich A, Mikheenko A, Shlemov A, Korobeynikov A, Mohimani H, Pevzner PA (2018). Increased diversity of peptidic natural products revealed by modification-tolerant database search of mass spectra. Nat Microbiol.

[23] Ricart E, Pupin M, Müller M, Lisacek F (2020). Automatic annotation and dereplication of tandem mass spectra of peptidic natural products. Anal Chem.

[24] Caboche S, Pupin M, Leclere V, Fontaine A, Jacques P, Kucherov G (2008). NORINE: a database of nonribosomal peptides. Nucleic Acids Res.

[25] Flissi A, Ricart E, Campart C, Chevalier M, Dufresne Y, Michalik J, Jacques P, Flahaut C, Lisacek F, Leclere V, Pupin M (2019). Norine: update of the nonribosomal peptide resource. Nucleic Acids Res.

[26] Flissi A, Dufresne Y, Michalik J, Tonon L, Janot S, Noe L, Jacques P, Leclere V, Pupin M (2016). Norine, the knowledgebase dedicated to non-ribosomal peptides, is now open to crowdsourcing. Nucleic Acids Res.

[27] Dufresne Y, Noe L, Leclere V, Pupin M (2015). Smiles2Monomers: a link between chemical and biological structures for polymers. J Cheminformatics.

[28] Ricart E, Leclere V, Flissi A, Mueller M, Pupin M, Lisacek F (2019). RBAN: Retro-biosynthetic analysis of nonribosomal peptides. J Cheminformatics.

[29] Novak, J.: CycloBranch (2021). https://ms.biomed.cas.cz/cyclobranch/. Accessed 12 Apr 2021

[30] Novak, J.: CycloBranch on GitHub (2021). https://github.com/novak-jiri/cyclobranch/. Accessed 12 Apr 2021

[31] Novak J, Skriba A, Havlicek V (2020). CycloBranch 2: molecular formula annotations applied to imzml data sets in bimodal fusion and LC-MS data files. Anal Chem.

[32] Behsaz B, Mohimani H, Gurevich A, Prjibelski A, Fisher M, Vargas F, Smarr L, Dorrestein PC, Mylne JS, Pevzner PA (2020). De Novo peptide sequencing reveals many cyclopeptides in the human gut and other environments. Cell Syst.

[33] Mohimani H, Pevzner PA (2016). Dereplication, sequencing and identification of peptidic natural products: From genome mining to peptidogenomics to spectral networks. Nat Prod Rep.

[34] Privratsky, J.: MassSpecBlocks: Database of Sequences and Building Blocks of Microbial Metabolites for Mass Spectra Analysis. Master’s Thesis, Czech Technical University in Prague, Faculty of Information Technology (2021). https://github.com/privrja/MassSpecBlocks/blob/main/text/MassSpecBlocks.pdf. Accessed 1 May 2021

[35] Privratsky, J.: MassSpecBlocks on GitHub (2021). https://github.com/privrja/MassSpecBlocks. Accessed 12 Apr 2021

[36] Facebook Inc.: React (2021). https://reactjs.org/. Accessed 12 Apr 2021

[37] OpenJS Foundation: Node.js (2021). https://nodejs.org/. Accessed 12 Apr 2021

[38] Kim S, Thiessen PA, Bolton EE, Bryant SH (2015). PUG-SOAP and PUG-REST: web services for programmatic access to chemical information in PubChem. Nucleic Acids Res.

[39] Royal Society of Chemistry: APIs (2021). https://developer.rsc.org/apis. Accessed 12 Apr 2021

[40] Van Santen, J.A., et al.: NP Atlas API (2021). https://www.npatlas.org/api/v1/docs. Accessed 17 Jun 2021

[41] EMBL EBI: ChEBI Web Services (2021). https://www.ebi.ac.uk/chebi/webServices.do. Accessed 12 Apr 2021

[42] Probst D, Reymond J-L (2018). SmilesDrawer: parsing and drawing SMILES-encoded molecular structures using client-side javascript. J Chem Inf Model.

[43] Bienfait B, Ertl P (2013). JSME: a free molecule editor in JavaScript. J Cheminformatics.

[44] Oracle Corporation and/or its affiliates: MySQL™ (2021). https://www.mysql.com/. Accessed 12 Apr 2021

[45] Symfony SAS: Symfony™ (2021). https://symfony.com/. Accessed 12 Apr 2021

[46] Privratsky, J.: MassSpecBlocks—REST API Documentation (2021). https://ms.biomed.cas.cz/msb-backend/public/index.php/rest/doc. Accessed 14 Jun 2021

[47] Weininger D, Weininger A, Weininger JL (1989). SMILES. 2. Algorithm for generation of unique SMILES notation. J Chem Inf Comput Sci.

[48] Pavlaskova K, Nedved J, Kuzma M, Zabka M, Sulc M, Sklenar J, Novak P, Benada O, Kofronova O, Hajduch M, Derrick PJ, Lemr K, Jegorov A, Havlicek V (2010). Characterization of pseudacyclins A-E, a suite of cyclic peptides produced by *Pseudallescheria boydii*. J Nat Prod.

[49] Rácz A, Bajusz D, Héberger K (2018). Life beyond the Tanimoto coefficient: Similarity measures for interaction fingerprints. J Cheminformatics.

[50] Privratsky, J., Novak, J.: MassSpecBlocks - Video Tutorial (2021). https://ms.biomed.cas.cz/msb/msb-tutorial.mp4. Accessed 19 Jun 2021

[51] Jegorov A, Paizs B, Zabka M, Kuzma M, Havlicek V, Giannakopulos AE, Derrick PJ (2003). Profiling of cyclic hexadepsipeptides roseotoxins synthesized in vitro and in vivo: A combined tandem mass spectrometry and quantum chemical study. Eur J Mass Spectrom.

[52] Pluhacek T, Skriba A, Novak J, Luptakova D, Havlicek V (2019). Analysis of microbial siderophores by mass spectrometry. Methods Mol Biol.

