# Supplementary material for: MassSpecBlocks: a web-based tool to create building blocks and sequences of nonribosomal peptides and polyketides for tandem mass spectra analysis
Source: J Cheminform. 2021 Jul 7;13:51. doi: 10.1186/s13321-021-00530-2 (PMC8265115; doi:10.1186/s13321-021-00530-2)
Supplement: Supplementary file 1 — Additional file 1. Additional figures. [file 13321_2021_530_MOESM1_ESM.pdf]

Additional file 1

MassSpecBlocks: A Web-based Tool to Create Building Blocks and Sequences of Nonribosomal Peptides and Polyketides for Tandem Mass Spectra Analysis

Jan Přívratský[1] and Jiří Novák[1,2,*]

[1]Faculty of Information Technology, Czech Technical University in Prague, Thákurova 9, 160 00 Prague, Czech Republic
[2]Institute of Microbiology, Czech Academy of Sciences, Vídeňská 1083, 142 20 Prague, Czech Republic
[*]e-mail: jiri.novak@biomed.cas.cz

Create new block

Name: Name Acronym: Acronym Formula: Residue SMILES: SMILES Family: Select... Editor

Create new block

List of blocks - My Compounds - 18 rows

Name	Acronym	Residue	Mass	Losses	Family	SMILES	Identifier	Actions
Name	Acronym	Formula	Mass from<br>Mass to	Losses	Family	Smiles	Identifier	Filter Clear
3-Methyl-Proline	3Me-Pro	C6H9NO	111.068414			CC1CCNC1C(O)=O	CID: 14185042	Editor Show FindRef Clone Usage Delete
beta-Alanine	bAla	C3H5NO	71.037113			NCCC(O)=O	CID: 239	Editor Show FindRef Clone Usage Delete
4-Methyl-2-Hydroxy-Pentanoic Acid	C5:0-Me(4)-OH(2)	C6H10O2	114.068079			CC(C)CC(O)C(O)=O	CID: 92779	Editor Show FindRef Clone Usage Delete
3-Hydroxy-Octanoic Acid	C8:0-OH(3)	C8H14O2	142.099379			CCCCCC(O)CC(O)=O	CID: 26613	Editor Show FindRef Clone Usage Delete
Formyl-Hydroxy-Ornithine	Fo-OH-Orn	C6H10N2O3	158.069142			NC(CCCN(O)C=O)C(O)=O	CID: 75114616	Editor Show FindRef Clone Usage Delete
(-2H) N-Hydroxy-Cadaverine	Hpd	C5H12N2O	116.094963			NCCCCCNO	CID: 22119660	Editor Show FindRef Clone Usage Delete
Isoleucine	Ile	C6H11NO	113.084064			CCC(C)C(N)C(O)=O	CID: 791	Editor Show FindRef Clone Usage Delete
N-Acetyl-Isoleucine	NAc-Ile	C8H13NO2	155.094628			CCC(C)C(NC(C)=O)C(O)=O	CID: 306109	Editor Show FindRef Clone Usage Delete
N-Methyl-Alanine	NMe-Ala	C4H7NO	85.052764			CNC(C)C(O)=O	CID: 4377	Editor Show FindRef Clone Usage Delete
N-Methyl-Valine	NMe-Val	C6H11NO	113.084064			CNC(C(C)C)C(O)=O	CID: 4378	Editor Show FindRef Clone Usage Delete
Hydroxy-Aspartic acid	OH-Asp	C4H5NO4	131.021857			NC(C(O)C(O)=O)C(O)=O	CID: 5425	Editor Show FindRef Clone Usage Delete
Hydroxy-Ornithine	OH-Orn	C5H10N2O2	130.074227			NC(CCCNO)C(O)=O	CID: 22326348	Editor Show FindRef Clone Usage Delete
Ornithine	Orn	C5H10N2O	114.079313			NCCCC(N)C(O)=O	CID: 389	Editor Show FindRef Clone Usage Delete
Phenylalanine	Phe	C9H9NO	147.068414			NC(CC1=CC=CC=C1)C(O)=O	CID: 994	Editor Show FindRef Clone Usage Delete
Proline	Pro	C5H7NO	97.052764			OC(=O)C1CCCN1	CID: 614	Editor Show FindRef Clone Usage Delete
(-2H) putrescine	Put	C4H10N2	86.084398			NCCCCN	CID: 1045	Editor Show FindRef Clone Usage Delete
Serine	Ser	C3H5NO2	87.032028			NC(CO)C(O)=O	CID: 617	Editor Show FindRef Clone Usage Delete
Succinic semialdehyde	Suc	C4H4O2	84.021129			OC(=O)CCC=O	CID: 1112	Editor Show FindRef Clone Usage Delete

Figure S1. A sample list of building blocks used in this study.

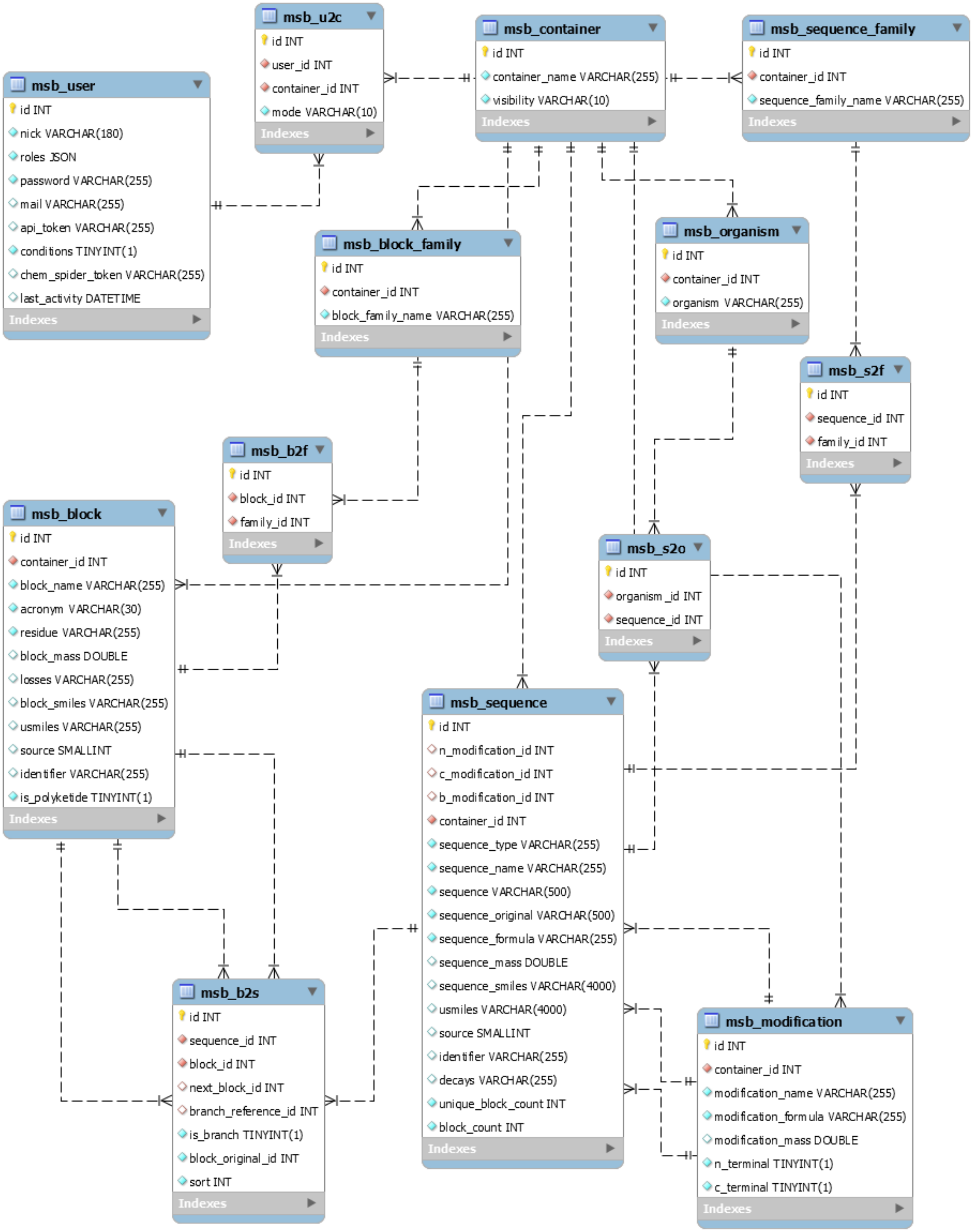

Figure S2. Data model (a detailed description of individual attributes is available in the project's repository https://github.com/privrja/MassSpecBlocks/).

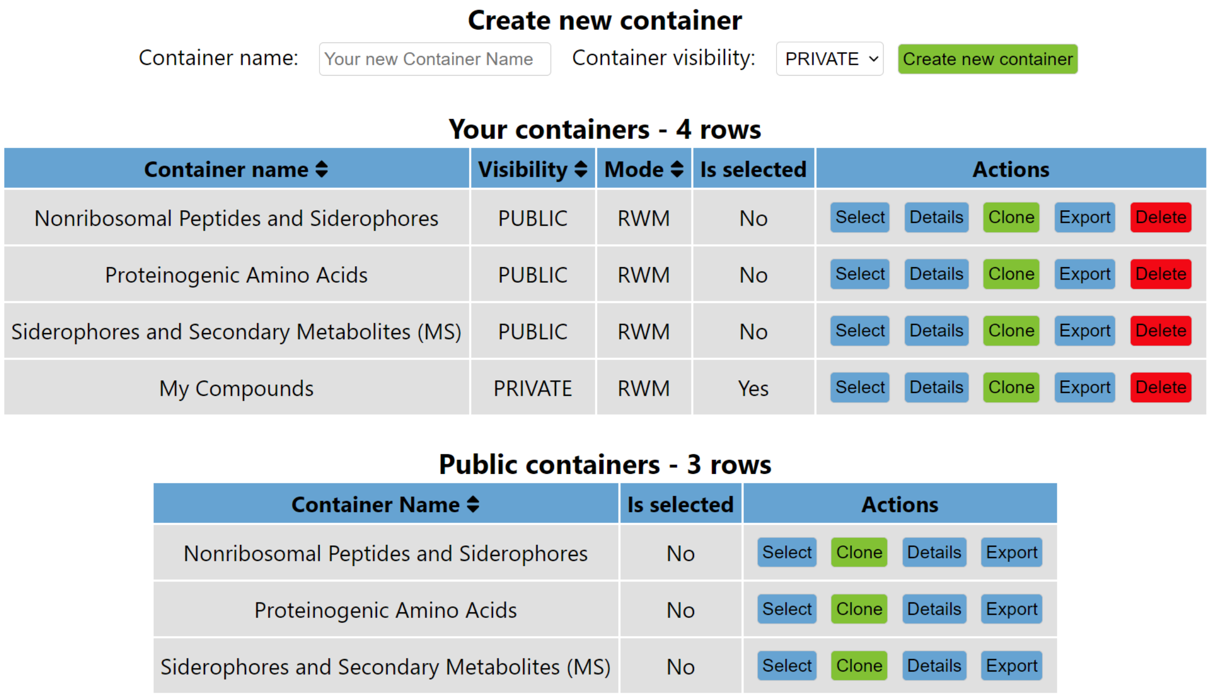

Figure S3. A list of containers.

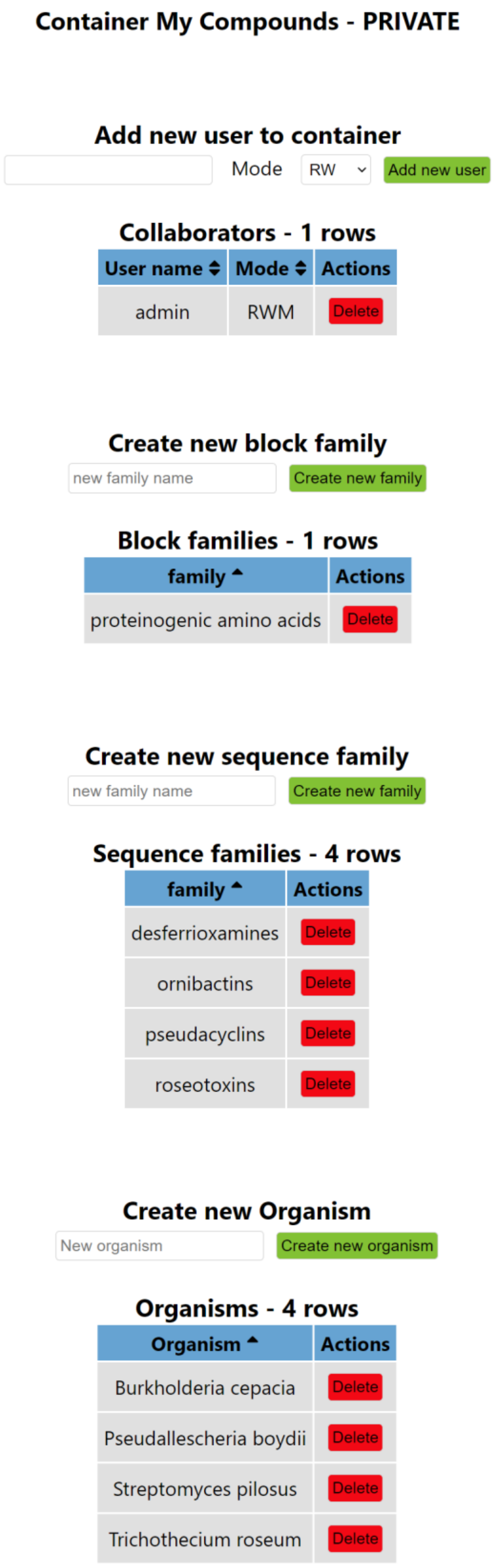

Figure S4. Configuration of a container (accessible using the “Details” button next to the container’s name in the list of containers).

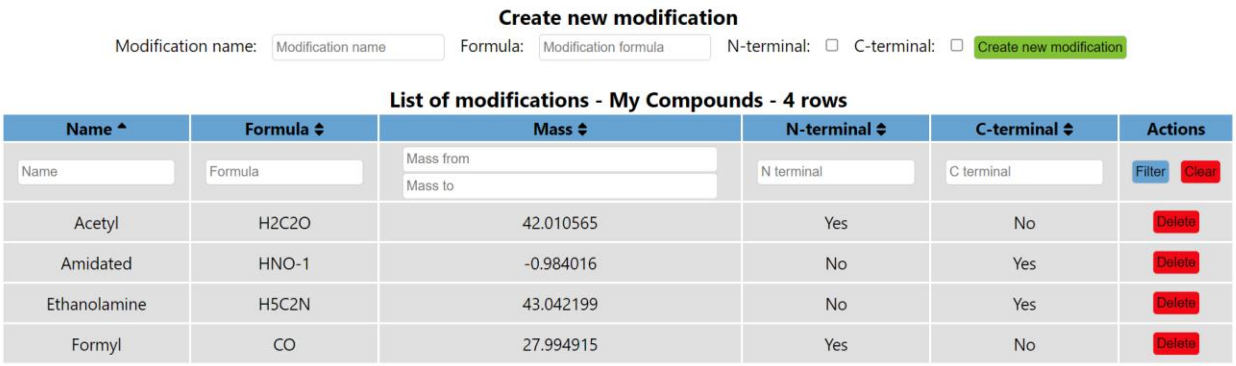

Figure S5. A list of terminal modifications.

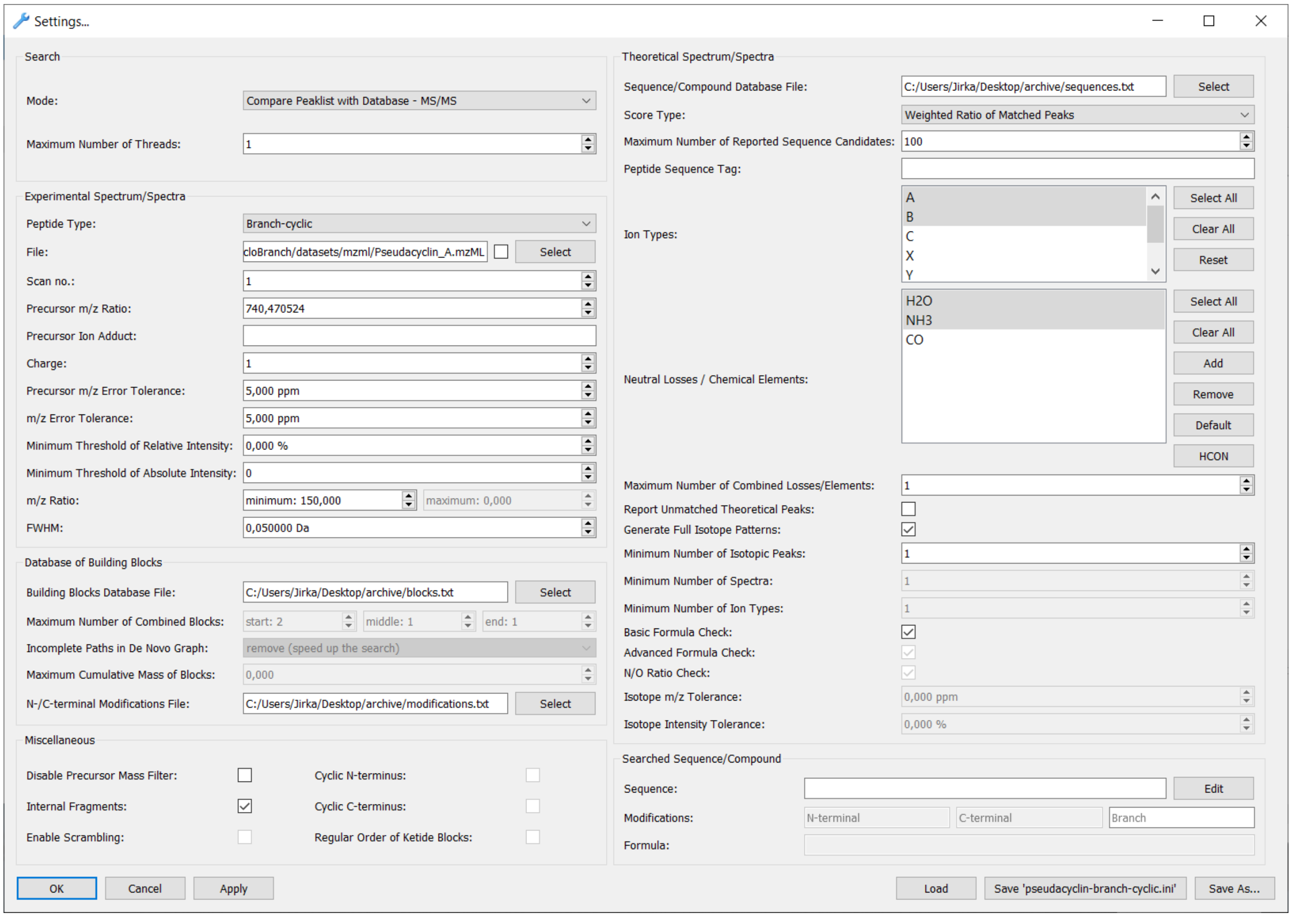

Figure S6. CycloBranch's settings dialog – the experimental mass spectrum of pseudacyclin A was compared with theoretical mass spectra generated from an NPR/PK sequence database.

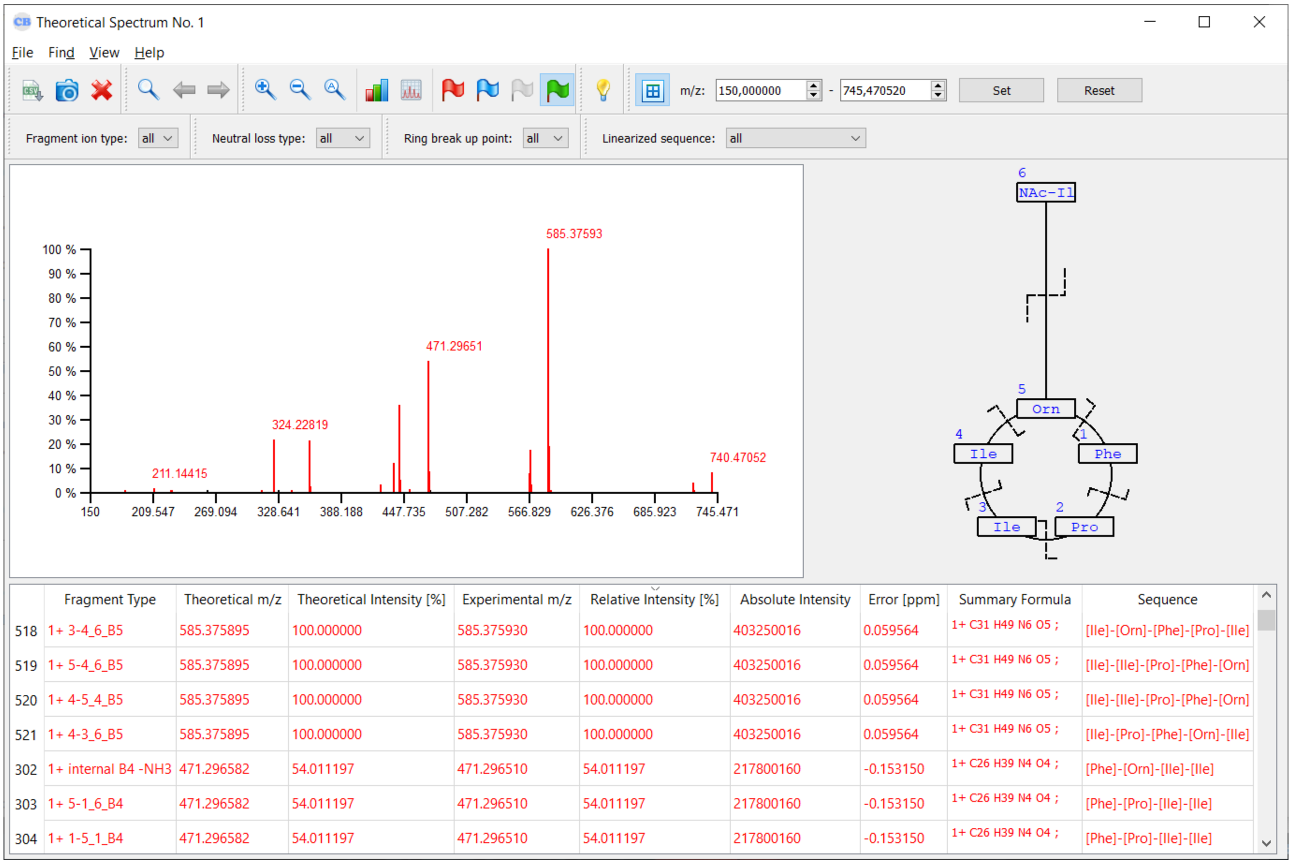

	Fragment Type	Theoretical m/z	Theoretical Intensity [%]	Experimental m/z	Relative Intensity [%]	Absolute Intensity	Error [ppm]	Summary Formula	Sequence
518	1+ 3-4_6_B5	585.375895	100.000000	585.375930	100.000000	403250016	0.059564	1+ C31 H49 N6 O5 ;	[Ile]-[Orn]-[Phe]-[Pro]-[Ile]
519	1+ 5-4_6_B5	585.375895	100.000000	585.375930	100.000000	403250016	0.059564	1+ C31 H49 N6 O5 ;	[Ile]-[Ile]-[Pro]-[Phe]-[Orn]
520	1+ 4-5_4_B5	585.375895	100.000000	585.375930	100.000000	403250016	0.059564	1+ C31 H49 N6 O5 ;	[Ile]-[Ile]-[Pro]-[Phe]-[Orn]
521	1+ 4-3_6_B5	585.375895	100.000000	585.375930	100.000000	403250016	0.059564	1+ C31 H49 N6 O5 ;	[Ile]-[Pro]-[Phe]-[Orn]-[Ile]
302	1+ internal B4 -NH3	471.296582	54.011197	471.296510	54.011197	217800160	-0.153150	1+ C26 H39 N4 O4 ;	[Phe]-[Orn]-[Ile]-[Ile]
303	1+ 5-1_6_B4	471.296582	54.011197	471.296510	54.011197	217800160	-0.153150	1+ C26 H39 N4 O4 ;	[Phe]-[Pro]-[Ile]-[Ile]
304	1+ 1-5_1_B4	471.296582	54.011197	471.296510	54.011197	217800160	-0.153150	1+ C26 H39 N4 O4 ;	[Phe]-[Pro]-[Ile]-[Ile]

Figure S7. Annotated mass spectrum of pseudacyclin A in CycloBranch.

a)

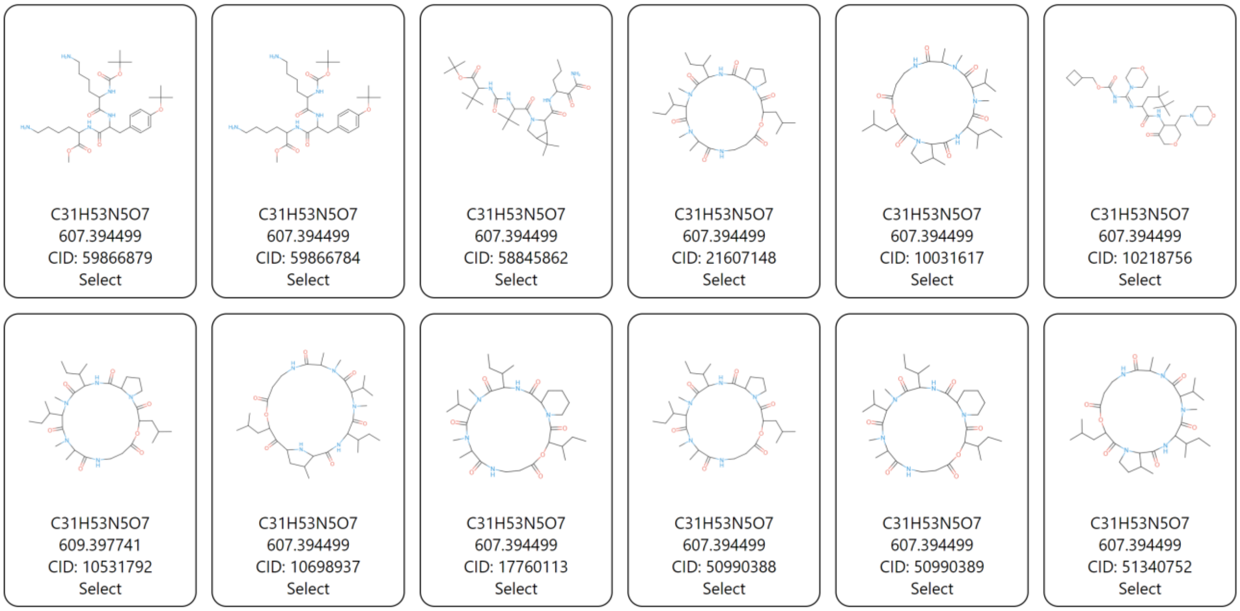

b)

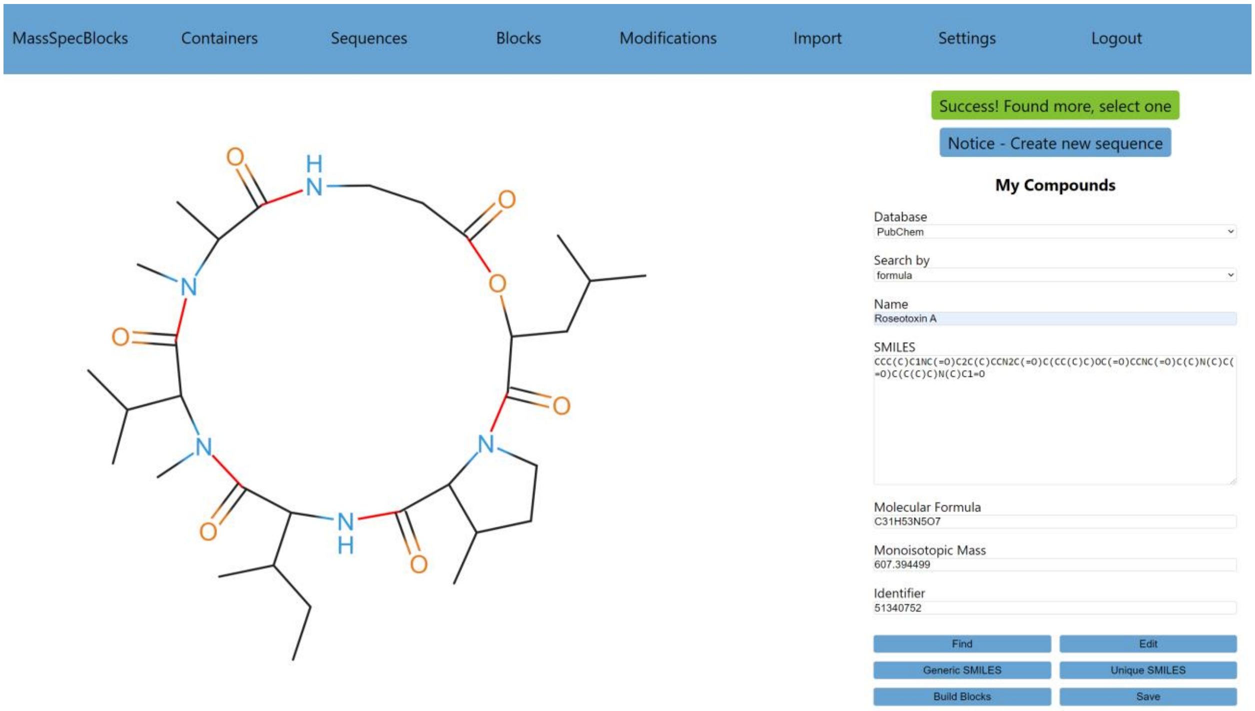

Figure S8. a) The molecular formula $C_{31}H_{53}N_5O_7$ was searched in PubChem. Sixty-six compounds were reported by MassSpecBlocks (a shortened list is shown), and roseotoxin A (CID: 51340752) was selected for further analysis. b) Annotation of peptide bonds and an ester bond in roseotoxin A.

Sequence - 6 blocks

Type: cyclic   Sequence: [Ile]-[NMe-Val]-[NMe-Ala]-[bAla]-[C5:0-Me(4)-OH(2)]-[3Me-Pro]

Family: roseotoxins ×   Organism: Trichothecium roseum ×

Edit same blocks together

MSB acronym	Preview	Acronym	SMILES	Name	Formula	Mass	Losses	Identifier	Actions
Ile	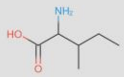	Ile	CCC(C)C(N)C(O)=O	Isoleucine	C6H11NO	113.084064		CID: 791	Editor FindRef Remove
NMe-Val	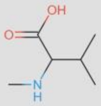	NMe-Val	CNC(C(C)C)C(O)=O	N-Methyl-Valine	C6H11NO	113.084064		CID: 4378	Editor FindRef Remove
NMe-Ala	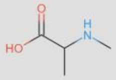	NMe-Ala	CNC(C)C(O)=O	N-Methyl-Alanine	C4H7NO	85.052764		CID: 4377	Editor FindRef Remove
bAla	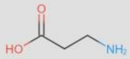	bAla	NCCC(O)=O	beta-Alanine	C3H5NO	71.037113		CID: 239	Editor FindRef Remove
C5:0-Me(4)-OH(2)	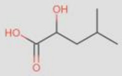	C5:0-Me(4)-OH(2)	CC(C)CC(O)C(O)=O	4-Methyl-2-Hydroxy-Pentanoic Acid	C6H10O2	114.068079		CID: 92779	Editor FindRef Remove
3Me-Pro	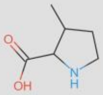	3Me-Pro	CC1CCNC1C(O)=O	3-Methyl-Proline	C6H9NO	111.068414		CID: 14185042	Editor FindRef Remove

Figure S9. Building blocks of roseotoxin A.

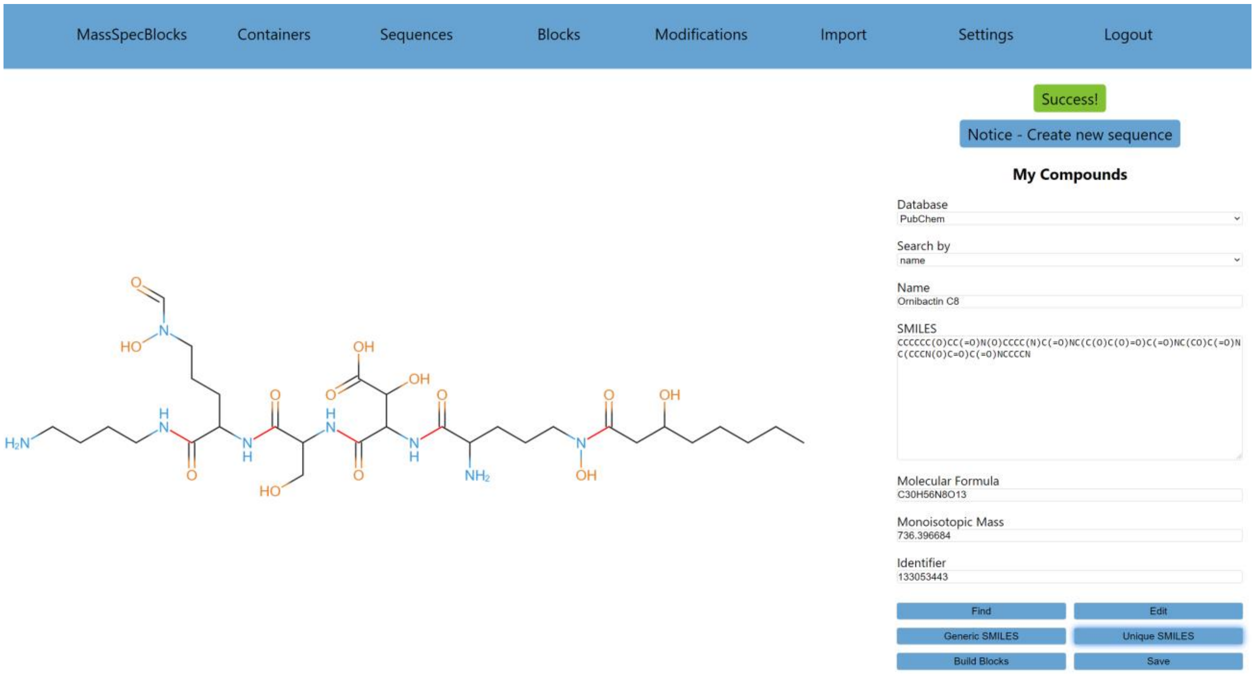

Figure S10. Annotation of peptide bonds in the linear PK siderophore ornibactin C8. The compound has a PK building block putrescine $NH_2(CH_2)_4NH_2$ attached at C-terminus.

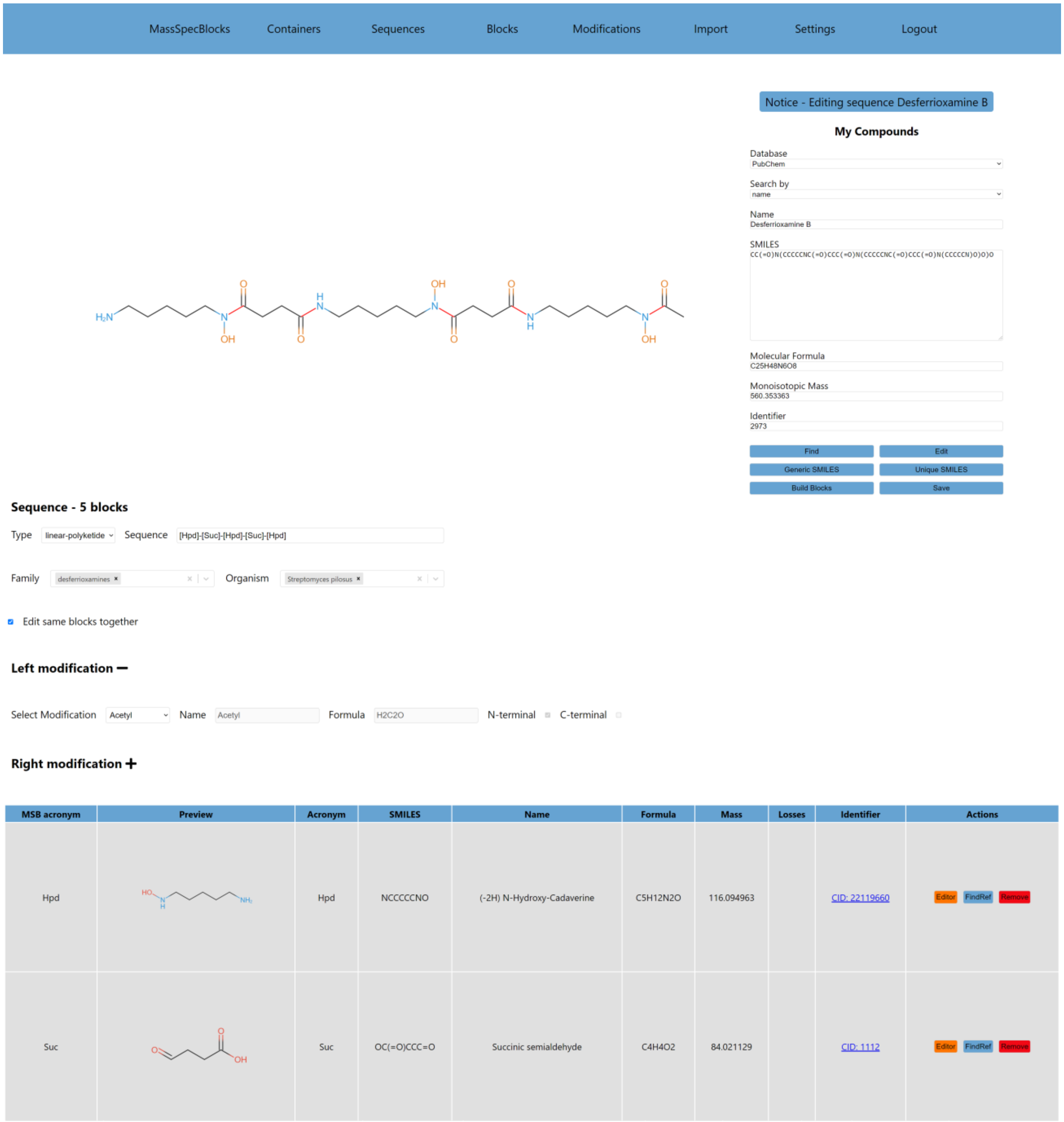

Figure S11. Decomposition of a linear PK siderophore desferrioxamine B into building blocks (shortened version).

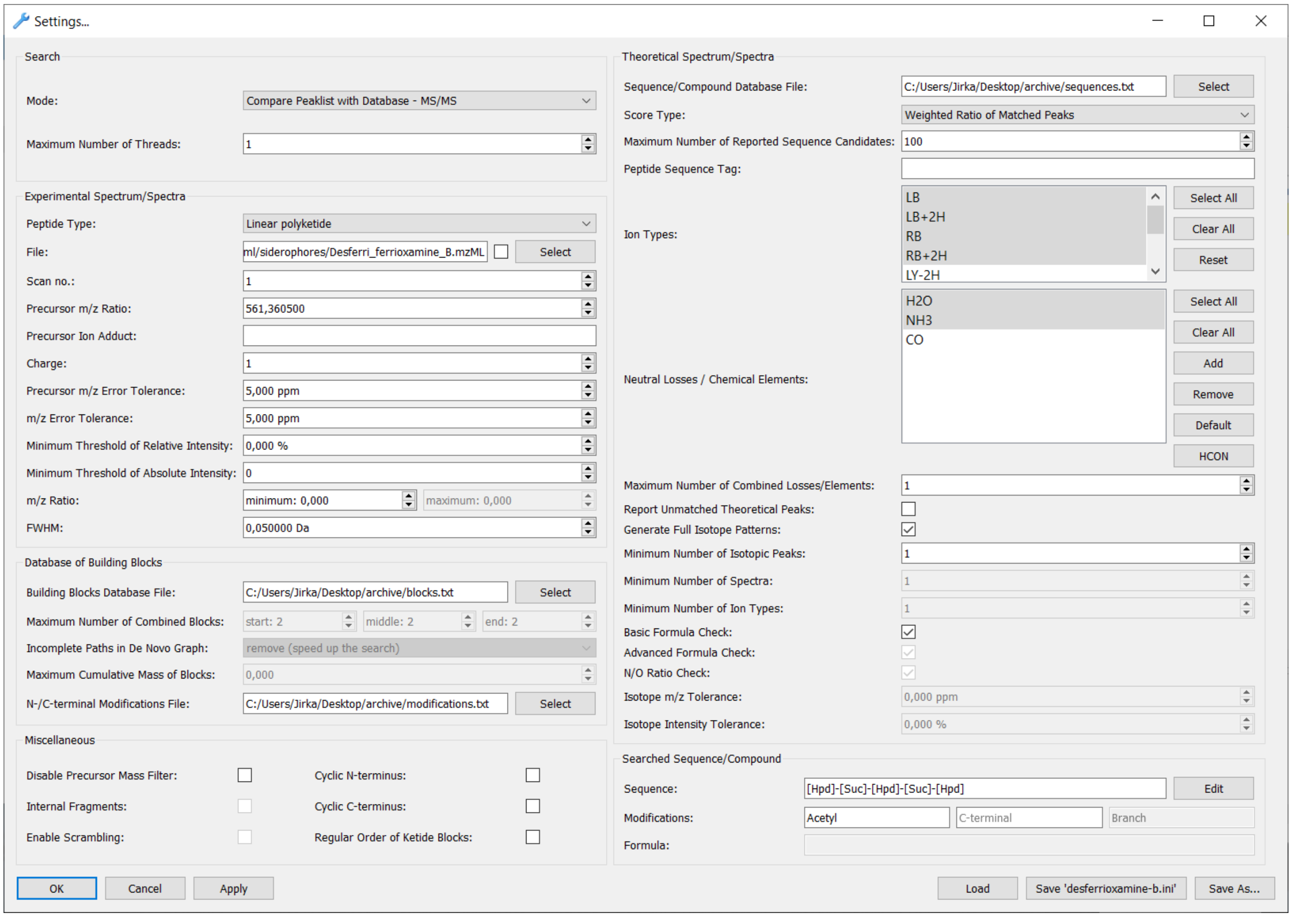

Figure S12. CycloBranch's settings dialog – the experimental mass spectrum of desferrioxamine B in mzML file format was compared with theoretical mass spectra generated from the sequence database exported from MassSpecBlocks. The input database file (sequences.txt) contained 146 NRPs and siderophore sequences. The list of building blocks (blocks.txt) included 85 items, and the list of terminal modifications had four items (modifications.txt).

a)

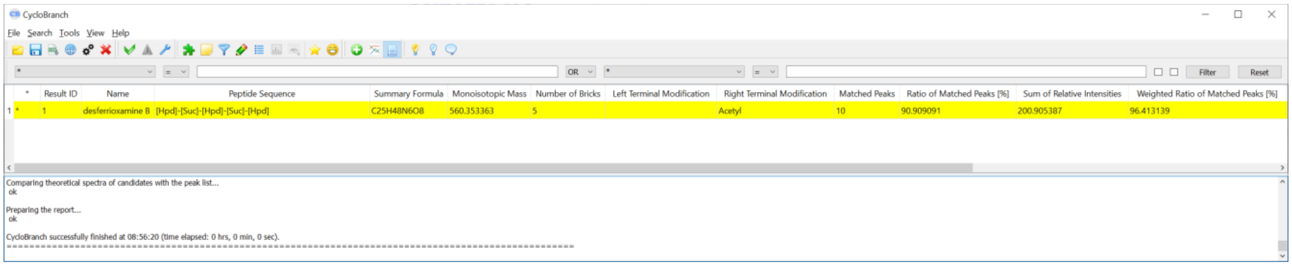

	*	Result ID	Name	Peptide Sequence	Summary Formula	Monoisotopic Mass	Number of Bricks	Left Terminal Modification	Right Terminal Modification	Matched Peaks	Ratio of Matched Peaks [%]	Sum of Relative Intensities	Weighted Ratio of Matched Peaks [%]
1	*	1	desferrioxamine B	[Hpd]-[Suc]-[Hpd]-[Suc]-[Hpd]	C25H48N6O8	560.353363	5		Acetyl	10	90.909091	200.905387	96.413139

b)

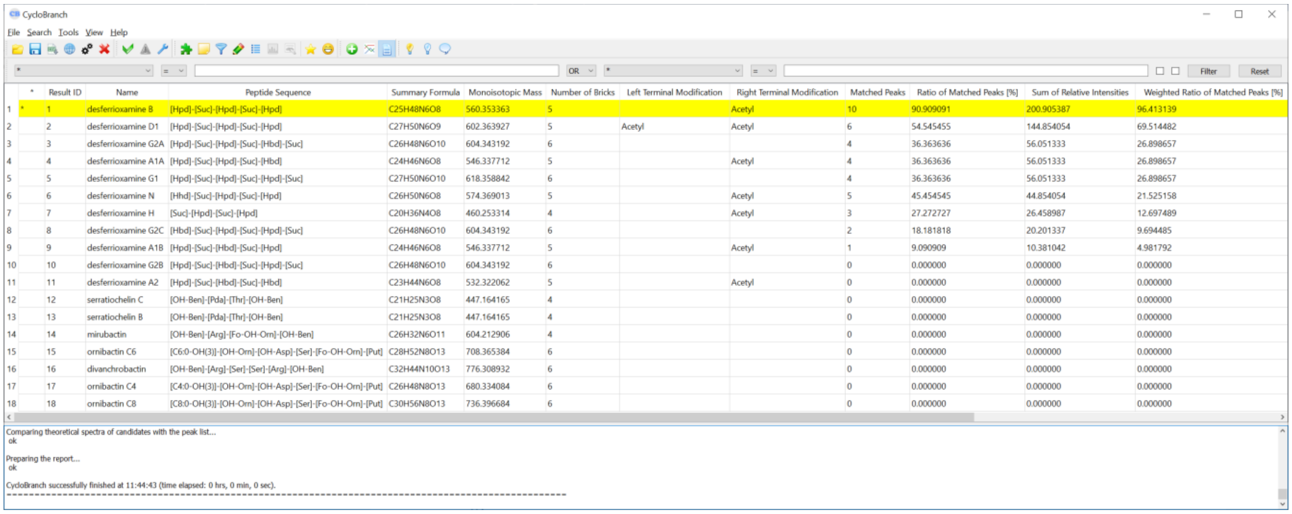

	*	Result ID	Name	Peptide Sequence	Summary Formula	Monoisotopic Mass	Number of Bricks	Left Terminal Modification	Right Terminal Modification	Matched Peaks	Ratio of Matched Peaks [%]	Sum of Relative Intensities	Weighted Ratio of Matched Peaks [%]
1	*	1	desferrioxamine B	[Hpd]-[Suc]-[Hpd]-[Suc]-[Hpd]	C25H48N6O8	560.353363	5		Acetyl	10	90.909091	200.905387	96.413139
2		2	desferrioxamine D1	[Hpd]-[Suc]-[Hpd]-[Suc]-[Hpd]	C27H50N6O9	602.363927	5	Acetyl	Acetyl	6	54.545455	144.854054	69.514482
3		3	desferrioxamine G2A	[Hpd]-[Suc]-[Hpd]-[Suc]-[Hbd]-[Suc]	C26H48N6O10	604.343192	6			4	36.363636	56.051333	26.898657
4		4	desferrioxamine A1A	[Hpd]-[Suc]-[Hpd]-[Suc]-[Hbd]	C24H46N6O8	546.337712	5		Acetyl	4	36.363636	56.051333	26.898657
5		5	desferrioxamine G1	[Hpd]-[Suc]-[Hpd]-[Suc]-[Hpd]-[Suc]	C27H50N6O10	618.358842	6			4	36.363636	56.051333	26.898657
6		6	desferrioxamine N	[Hhd]-[Suc]-[Hpd]-[Suc]-[Hpd]	C26H50N6O8	574.369013	5		Acetyl	5	45.454545	44.854054	21.525158
7		7	desferrioxamine H	[Suc]-[Hpd]-[Suc]-[Hpd]	C20H36N4O8	460.253314	4		Acetyl	3	27.272727	26.458987	12.697489
8		8	desferrioxamine G2C	[Hbd]-[Suc]-[Hpd]-[Suc]-[Hpd]-[Suc]	C26H48N6O10	604.343192	6			2	18.181818	20.201337	9.694485
9		9	desferrioxamine A1B	[Hpd]-[Suc]-[Hbd]-[Suc]-[Hpd]	C24H46N6O8	546.337712	5		Acetyl	1	9.090909	10.381042	4.981792
10		10	desferrioxamine G2B	[Hpd]-[Suc]-[Hbd]-[Suc]-[Hpd]-[Suc]	C26H48N6O10	604.343192	6			0	0.000000	0.000000	0.000000
11		11	desferrioxamine A2	[Hpd]-[Suc]-[Hbd]-[Suc]-[Hbd]	C23H44N6O8	532.322062	5		Acetyl	0	0.000000	0.000000	0.000000
12		12	serratiochelin C	[OH-Ben]-[Pda]-[Thr]-[OH-Ben]	C21H25N3O8	447.164165	4			0	0.000000	0.000000	0.000000
13		13	serratiochelin B	[OH-Ben]-[Pda]-[Thr]-[OH-Ben]	C21H25N3O8	447.164165	4			0	0.000000	0.000000	0.000000
14		14	mirubactin	[OH-Ben]-[Arg]-[Fo-OH-Orn]-[OH-Ben]	C26H32N6O11	604.212906	4			0	0.000000	0.000000	0.000000
15		15	ornibactin C6	[C6:0-OH(3)]-[OH-Orn]-[OH-Asp]-[Ser]-[Fo-OH-Orn]-[Put]	C28H52N8O13	708.365384	6			0	0.000000	0.000000	0.000000
16		16	divanchrobactin	[OH-Ben]-[Arg]-[Ser]-[Ser]-[Arg]-[OH-Ben]	C32H44N10O13	776.308932	6			0	0.000000	0.000000	0.000000
17		17	ornibactin C4	[C4:0-OH(3)]-[OH-Orn]-[OH-Asp]-[Ser]-[Fo-OH-Orn]-[Put]	C26H48N8O13	680.334084	6			0	0.000000	0.000000	0.000000
18		18	ornibactin C8	[C8:0-OH(3)]-[OH-Orn]-[OH-Asp]-[Ser]-[Fo-OH-Orn]-[Put]	C30H56N8O13	736.396684	6			0	0.000000	0.000000	0.000000

Figure S13. CycloBranch's output window; the experimental spectrum of desferrioxamine B was compared with theoretical spectra – a) if the precursor mass filter was enabled in the settings dialog, the desferrioxamine B was reported as a single hit; b) if the precursor filter was disabled, the desferrioxamine B was still reported as the top hit (the weighted ratio of matched peaks was used as the scoring function and the value was 96.4% for the first sequence).

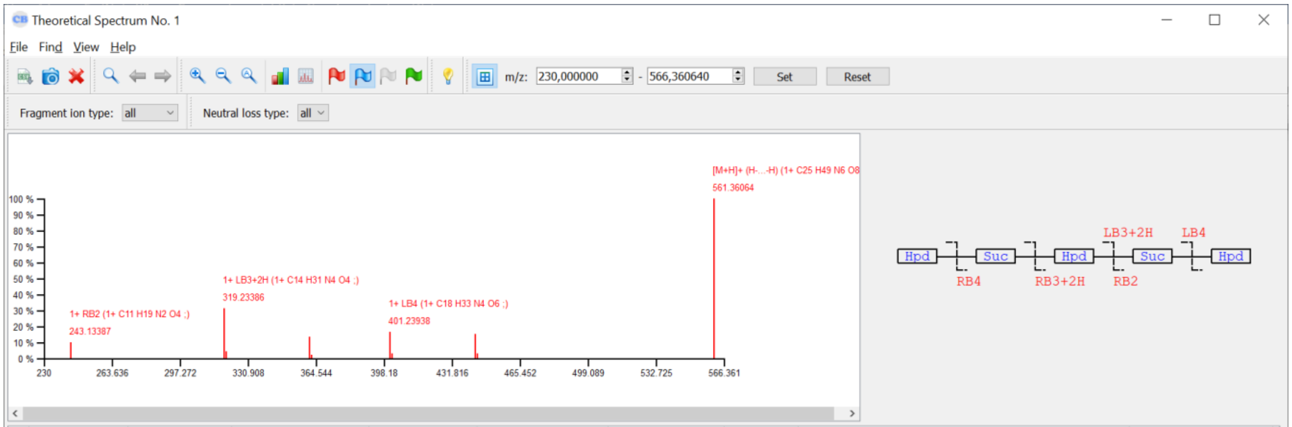

	Fragment Type	Theoretical m/z	Theoretical Intensity [%]	Experimental m/z	Relative Intensity [%]	Absolute Intensity	Error [ppm]	Summary Formula	Sequence
1	1+ RB2	243.133934	10.381042	243.133870	10.381042	120772712	-0.261282	1+ C11 H19 N2 O4 ;	[Hpd]-[Suc]
4	1+ LB3+2H	319.233982	31.447321	319.233860	31.447321	365857152	-0.381921	1+ C14 H31 N4 O4 ;	[Hpd]-[Suc]-[Hpd]
5	1+ LB3+2H	320.236860	5.386413	320.237230	4.402675	51220584	1.154538	1+ 13C C13 H31 N4 O4 [88.40%];1+ C14 H31 15N N3 O4 [8.63%];1+ C14 2H H30	[Hpd]-[Suc]-[Hpd]
8	1+ RB3+2H	361.244547	13.800030	361.244470	13.800030	160549120	-0.212068	1+ C16 H33 N4 O5 ;	[Hpd]-[Suc]-[Hpd]
9	1+ RB3+2H	362.247485	2.670666	362.247810	2.277915	26501190	0.897416	1+ 13C C15 H33 N4 O5 [89.42%];1+ C16 H33 15N N3 O5 [7.63%];1+ C16 2H H32	[Hpd]-[Suc]-[Hpd]
12	1+ LB4	401.239461	16.796589	401.239380	16.796589	195410992	-0.202449	1+ C18 H33 N4 O6 ;	[Hpd]-[Suc]-[Hpd]-[Suc]
13	1+ LB4	402.242444	3.620311	402.242720	3.404748	39610728	0.687109	1+ 13C C17 H33 N4 O6 [90.32%];1+ C18 H33 15N N3 O6 [6.85%];1+ C18 2H H32	[Hpd]-[Suc]-[Hpd]-[Suc]
16	1+ RB4	443.250026	15.226324	443.249940	15.226324	177142576	-0.193834	1+ C20 H35 N4 O7 ;	[Hpd]-[Suc]-[Hpd]-[Suc]
17	1+ RB4	444.253047	3.620529	444.253270	3.168743	36865056	0.501145	1+ 13C C19 H35 N4 O7 [90.97%];1+ C20 H35 15N N3 O7 [6.21%];1+ C20 2H H34	[Hpd]-[Suc]-[Hpd]-[Suc]
20	[M+H]+ (H-...-H)	561.360639	100.000000	561.360640	100.000000	1163396864	0.001784	1+ C25 H49 N6 O8 ;	

Figure S14. Annotated mass spectrum of desferrioxamine B in CycloBranch.
